# Optimal control under safety constraints and disturbances: a multi-step, off-policy adaptive dynamic programming approach

**DOI:** 10.1007/s11071-025-11329-3

**Published:** 2025-06-15

**Authors:** Jun Ye, Xiaowei Zhao, Yougang Bian, Manjiang Hu, Hongyang Dong

**Affiliations:** 1 State Key Laboratory of Advanced Design and Manufacturing Technology for Vehicle, College of Mechanical and Vehicle Engineering, Hunan University, Changsha, 410012 China; 2 The Intelligent Control and Smart Energy (ICSE) Research Group, School of Engineering, University of Warwick, Coventry, CV4 7AL UK

**Keywords:** Adaptive dynamic programming, Zero-sum game, Optimal control, Constrained control, Reinforcement learning

## Abstract

This paper introduces a multi-step, off-policy adaptive dynamic programming approach, in both model-free and model-based variants, intending to solve optimal control problems under disturbances and safety constraints. To provide a more accurate estimation of the performance function in the policy evaluation step, we employ an interleaved training method in the model-free scheme and utilize a prior model in the model-based version to mitigate the underestimation issue of the accumulated utility function. To further counteract the underestimation of the terminal performance function, dual critic neural networks are utilized. Additionally, to ensure a well-balanced trade-off between safety and performance requirements, the original unconstrained policy improvement process is transformed into a constrained optimization task with a far-sighted safety function. Furthermore, an actor-critic-disturbance framework is designed to handle safety constraints during the zero-sum game process, in which the disturbance policy and the performance function are alternately updated during the PEV step. Based on this, a rigorous theoretical analysis is conducted to evaluate the convergence property of the proposed method. Finally, simulation results and practical experiments demonstrate the effectiveness and safety of the proposed method.

## Introduction

Driven by pressing industrial needs, optimal control techniques have seen rapid advancements in recent years. Particularly, dynamic programming (DP) has attracted extensive attention due to its capacity to handle optimization and optimal control tasks through multi-step decision-making processes. However, DP faces significant challenges due to the ‘curse of dimensionality’—an issue characterized by exponential growth in computational complexity and resource demands with the increase in the number of dimensions (variables) of a problem [1]. In response to this issue, the adaptive dynamic programming (ADP) approach is proposed by integrating reinforcement learning and control theory, and this method has demonstrated its viability and effectiveness in tackling complex optimal control problems [2–4].

Various ADP schemes have been formulated in recent years, with policy iteration (PI) and value iteration (VI) emerging as two predominant algorithms within the ADP paradigm. For VI approaches, convergence necessitates the initial performance function to be set at zero. However, there is no specific requirement for the initially executed policy—it can be arbitrary [5]. In contrast, the PI method does not necessitate setting the performance function to zero but requires an admissible initial control policy before iterations. In typical PI designs, the iterated policy can consistently maintain admissibility [6, 7] when initiated from an admissible control law. This attribute makes the PI method a favored choice for practical real-time applications [8, 9].

On-policy and off-policy are two distinct training schemes in ADP [10]. The primary difference between them resides in action selection. Specifically, in an on-policy approach, the target policy set for improvement and the behavior policy used for action selection are identical. In contrast, these policies differ in an off-policy approach. As a result, the on-policy approach can be seen as a specific subtype of the off-policy method, usually characterized by a faster convergence process. This is attributable to the immediate updating of behavior, which is used for goal-directed interaction with the environment. However, obtaining the best control policy via an on-policy scheme is often challenging due to incomplete data [11]. In contrast, the off-policy method allows the use of diverse historical behaviors, significantly increasing data richness and achieving a balance between exploration and exploitation. This bolsters the applicability of off-policy approaches [12–14].

Multi-step schemes have garnered considerable attention in the pursuit of faster convergence and improved performance in ADP designs. These schemes, when compared to one-step cases such as the commonly used temporal difference (TD) methods, are capable of leveraging more valuable cost information from the training data. For instance, in [15], an on-policy multi-step heuristic dynamic programming (MsHDP) is proposed to expedite iteration based on the model information and alleviate the requirement for an initial admissible control law. However, the inadequate data utilization in the on-policy multi-step scheme may lead to unsatisfactory outcomes, and adopting persistent excitation during the training process could result in inevitable bias [16]. In comparison to the on-policy multi-step scheme, proposing an off-policy multi-step scheme is relatively more attractive. For instance, Kristopher [17] proposed a multi-step action-value algorithm $Q(\sigma )$, in which the sampling parameter $\sigma$ is introduced to unify full sampling and pure expectation. Notably, the algorithm $Q(\sigma )$ can be applied in both on-policy and off-policy schemes. In [18], a composite $Q$-learning framework is proposed to enhance data efficiency and evaluation accuracy by combining a short-term $Q$-function executed on a specific horizon and a long-term $Q$-function implemented on the remainder of the trajectory. However, the intractable inaccurate estimation of the performance function in these multi-step off-policy schemes can lead to extremely unstable training process, ultimately deteriorating results [19]. Therefore, in this study, an embedded training mode is proposed to eliminate underestimation.

In practical control applications, ensuring safety is paramount. Various types of constraints can be employed to guarantee safety during the control process. For instance, to protect actuators, action constraints can be implemented by designing a nonquadratic utility function [20, 21] or using saturated functions to limit the range of control policies. Addressing state constraints using existing ADP schemes is more challenging than handling control input constraints. Nonetheless, keeping states within a safe region is essential, particularly for safety–critical systems. Incorporating a reasonable penalty term into the cost function is a common method for restricting constrained states to the safe region [22, 23]. However, such designs break the optimal control structure. They can lead to bad transient performance and significant control costs when constrained states are close to the safe boundary. Moreover, this type of algorithm does not have the ability to recover from outside the safety boundary. Apart from incorporating penalty terms into the cost function, the control barrier function (CBF) method has attracted considerable attention due to its ability to balance between control performance and system safety. In [24], a safety–critical control algorithm is developed based on $Q$-learning and iterative ADP methods, in which discrete-time CBFs with novel definition of the safe set are introduced into the utility function for guaranteeing safety. In [25], Wang introduced a learning-based composite obstacle avoidance method that adeptly combines adaptive reinforcement learning with barrier functions in cost calculation, balancing policy optimization and real-time obstacle avoidance, while enhancing learning through simulated experience extrapolation. In reference to the composite avoidance control scheme proposed in [25], Ref. [26] extended it to multiplayer systems under threats of multiple no-entry or unsafe regions, and the proposed adaptive learning laws based on state-following kernel function have been demonstrated to effectively approximate Nash equilibrium solutions. Although CBFs offer excellent safety restrictions, designing an appropriate CBF is generally challenging when multiple state constraints are present. Furthermore, constraining states using CBFs necessitates model information, making this method inapplicable in many scenarios.

Aside from safety constraints, another significant issue that has garnered extensive attention in real-world applications is the presence of external disturbances. These disturbances can cause a mismatch between the real controller and the optimal controller due to the implementation of inaccurate dynamic models. As a result, addressing the negative impact of external disturbances is worth investigating. In [27], a novel deterministic policy gradient ADP method is developed for zero-sum game problems based on a periodic update approach. Different from conventional ADP methods, an advanced $Q$-learning algorithm is proposed by incorporating both the control input and the disturbance signal into the tracking error, which obviates the quadratic form of control and disturbance inputs directly [28]. In response to the challenge of uncertainty in cyber–physical systems, Jiang [29] developed a novel intelligent secure control scheme by integrating optimal control and zero-sum theory. This approach utilizes both PI and VI methods to solve the Hamilton–Jacobi–Isaacs equations, effectively mitigating the impact of actuator attacks and unmatched perturbation. To balance safety and control performance, Song [30] proposed an online optimal ${H}_{\infty}$ controller by introducing a barrier-actor-critic framework based on a transformation system designed to handle input and state constraints. In [31], an integral reinforcement learning algorithm is applied to derive a safe control policy by introducing a dynamic compensator and transforming the constrained safe control problem into an unconstrained optimal regulation problem. However, the construction of an unconstrained system and the transformation of state variables require extra efforts to ensure the fulfillment of state constraints [32].

In this paper, we aim to confront these aforementioned challenges by developing a multi-step, off-policy, safe ADP (SADP) scheme. It targets at solving optimal control problems while dealing with safety constraints and disturbances. The main contributions can be summarized as follows:

1. *Handling constraints:* In the pursuit of an optimal control policy, both state and control-input constraints are considered within policy improvement (PIM) process during each iteration. Regarding the handling of state constraints, a single inequality related to a far-sighted safe function is established to aggregately represent state constraints, which is then integrated with the objective function to reconstruct a constrained policy optimization process during each PIM. In managing control input constraints, a satisfied saturation function is implemented to effectively regulate the control policy.
2. *Dealing with disturbances:* To handle optimal control problems under disturbances while considering safe constraints, the proposed scheme diverges from the traditional actor-critic-disturbance framework [6, 14, 30]. It uniquely incorporates safety considerations persistently into the interplay between disturbance and control policies. Consequently, the adverse influence of disturbances, which can potentially cause the control object to deviate from the safety region, is constrained throughout the search for the optimal control policy. Meanwhile, to obtain excellent convergence conditions, the update of disturbance policy is integrated into the PEV process, and the convergence analysis based on this alternate iteration mode is provided.
3. *Mitigating underestimation:* To retain advantages associated with the implementation of the multi-step off-policy scheme, both model-based and model-free variants are proposed to mitigate the underestimation of the performance function in policy evaluation (PEV). Specifically, an interleaved manner between collection and training is introduced to eliminate the underestimation of the accumulated utility function in the model-free scheme; and the prior model information is utilized in the model-based scheme to achieve the same outcome. In addition, dual critic networks are introduced to alleviate the underestimation of the terminal function in both model-free and model-based schemes.

The rest of the paper is organized as follows. Section 2 presents the optimal control problem and formulates the control input and state constraints. In Sect. 3, the multi-step off-policy SADP scheme is derived, followed by analyses of the convergence properties of the proposed algorithm. Section 4 introduces implementation details of the proposed algorithm based on neural networks (NNs). In Sect. 5, simulation results and analyses are presented to demonstrate the effectiveness of the proposed method. Finally, Sect. 6 provides conclusions and outlines future work.

## Problem formulation

### Control problem formulation

We consider a typical class of nonlinear systems represented by the following form

1 $${x}_{t+1}=f\left({x}_{t},{u}_{t},{\omega}_{t}\right), t=\text{0,1},2,\dots$$

where ${x}_{t}\in {\mathbb{R}}^{m}$ represents the system state variable, ${u}_{t}\in {\mathbb{R}}^{n}$ denotes the control policy, ${\omega}_{t}\in {\mathbb{R}}^{n}$ is the disturbance item, and $m$ and $n$ denote the dimensions of state and policy spaces, respectively. The system functions $f\left(\bullet \right)$ is Lipschitz continuous within a compact set $\Omega$ that includes the origin point. Moreover, the system (1) is presumed to be stabilizable. Therefore, there always exists an admissible control law that can stabilize the system (1) asymptotically. In order to facilitate the analysis, the disturbance ${\omega}_{t}$ is assumed to be within a bound $\Vert {\omega}_{m}\Vert$, i.e., $\Vert {\omega}_{t}\Vert \le {\omega}_{m}$ and ${\omega}_{t}\in {\Psi}_{\omega}$, where ${\omega}_{m}$ is a positive constant and ${\Psi}_{\omega}$ is a set that contains all disturbance policies.

For the optimal control problem, the performance function can be constructed as

2 $${\mathbb{V}}\left({x}_{t}\right)=\sum_{l=t}^{\infty}{\gamma}^{l-t}U\left({x}_{l},{u}_{l},{\omega}_{l}\right),$$

where $0<\gamma \le 1$ represents a discount factor. In (2), the utility function, $U\left({x}_{t},{u}_{t},{\omega}_{t}\right)$, follows a quadratic form:

3 $$U\left({x}_{t},{u}_{t},{\omega}_{t}\right)={x}_{t}^{T}Q{x}_{t}+{u}_{t}^{T}R{u}_{t}-\beta {\omega}_{t}^{T}{\omega}_{t},$$

where $Q$ and $R$ are positive-definite weight matrices, $\beta$ is a positive constant representing the user-determined resistant degree of disturbances.

Based on (2) and Bellman’s optimality principle, the optimal performance function can be given by

4 $${\mathbb{V}}^{*}\left({x}_{t}\right)=\underset{{u}_{t}}{\text{min}}\underset{{\omega}_{t}}{\text{max}}\left(U\left({x}_{t},{u}_{t},{\omega}_{t}\right)+\gamma {\mathbb{V}}^{*}\left({x}_{t+1}\right)\right).$$

Next, the target is to find an optimal control policy, i.e.,

5 $${u}_{t}^{*}=\text{arg}\underset{{u}_{t}}{\text{min}}\left(U\left({x}_{t},{u}_{t},{\omega}_{t}\right)+\gamma {\mathbb{V}}^{*}\left({x}_{t+1}\right)\right).$$

Moreover, such an optimal control problem with disturbances can be regarded as a two-player zero-sum game, and the worst disturbance follows

6 $${\omega}_{t}^{*}=\text{arg}\underset{{\omega}_{t}}{\text{max}}\left(U\left({x}_{t},{u}_{t},{\omega}_{t}\right)+\gamma {\mathbb{V}}^{*}\left({x}_{t+1}\right)\right).$$

### Handling safe constraints

To obtain the optimal control policy (5) under disturbances, the traditional actor-critic-disturbance framework can be used [6, 27, 33, 34]. However, traditional actor-critic-disturbance framework’s limitations in handling safety constraints are mainly due to its reliance on gradient descent for policy improvement, a method inherently focused on unconstrained optimization. As a result, the optimization of control policy based on this method solely aims to optimize the performance function without considering safety constraints, which are crucial in safety–critical systems, such as spacecraft [35], autonomous system [36], and power system [37].

In this study, to achieve an excellent balance between optimality and control performance, control inputs are constrained by using a smooth and saturated function based on practical requirements. This means that the constrained control input is set boundedly, i.e., ${\Psi}_{u}=\left\{u|\left|{u}_{p}\right|\le {\overline{u}}_{p}, p=\text{1,2},\dots, n\right\}$, where ${\overline{u}}_{p}$ is the bound for $p$-th control input. In addition to control constraints, state constraints are also vital. Moreover, to address state constraints effectively, the policy update process is formulated as a constrained optimization problem. To this end, the cost function during handling state constraints, referred to as the safe function in our work, is defined in the following equation.

7 $${J}_{c}\left({x}_{c,t}^{{N}_{c}}\right)=\sum_{l=t}^{{t+N}_{c}-1}{\gamma}_{c}^{l-t}{U}_{c}\left({x}_{c,l}\right),$$

where ${x}_{c,t}$ denotes the constrained state at time $t$, ${U}_{c}\left({x}_{c,l}\right)$ represents the single step cost w.r.t the constrained state ${x}_{c,l}$. It is emphasized that the form of ${U}_{c}\left({x}_{c,l}\right)$ should be determined case-by-case based on different scenarios and requirements, instead of simply employing a quadratic form. In practice, executing a single step for constrained states may not yield a significant trade-off between safety and control performance. Therefore, the cumulative form of a multi-step cost function for constrained states is introduced in (7), where ${N}_{c}$ denotes the predicted step, and a discount factor is introduced, satisfying $0<{\gamma}_{c}\le 1$.

Based on (7), the state constraint can be expressed as

8 $${J}_{c}\left({x}_{c,t}^{{N}_{c}}\right)\le {d}_{c},$$

where ${d}_{c}$ denotes the safe boundary of the constrained state. Compared to the safe function of constrained states defined in [38, 39], the number of constraint inequalities in (8) depends only on the number of constrained states, rather than [${N}_{c}\times$ the number of constraints]. This can dramatically advance the computation speed and the feasibility of the whole control task.

Based on the handling of safe constraints presented above, the control object becomes to find a constrained optimal control policy that follows

9 $$\begin{aligned}&{u}_{t}^{*}=\text{arg}\underset{{u}_{t}}{\text{min}}\left(U\left({x}_{t},{u}_{t},{\omega}_{t}\right)+\gamma {\mathbb{V}}^{*}\left({X}_{t+1}\right)\right) \\& \text{s}.\text{t}.:{J}_{c}\left({x}_{c,t}^{{N}_{c}}\right)\le {d}_{c}.\end{aligned}$$

Taking into account the analyses above, we can now formalize the optimal control problem subject to safety constraints and disturbances. The task is to find an optimal control policy that can minimize the prescribed performance function (2) under the worst-case disturbance policy (6), while fulfilling safe constraints with the constrained PIM in (9). Unfortunately, traditional ADP schemes are unsuitable to solve such a complex constrained optimal control problem. To meet this challenge, we develop multi-step SADP approaches, with key details presented in Sect. 3.

#### Remark 1:

In addressing control input constraints within optimal control problems, existing methods offer varying degrees of effectiveness, such as achieving an explicit optimal control policy based on the non-quadratic utility [20, 34, 40] employing penalties or barriers on control variables, and applying Lagrange multipliers, but these approaches have certain limitations. For instance, the non-quadratic utility approach may face extreme challenges when dealing with scenarios where the traditional solution structure of the optimization problem changes, such as addressing state and control input constraints simultaneously in this study. Additionally, the implementation of penalties or barriers may struggle to ensure a balance between optimality and control performance. Furthermore, Lagrange multipliers may increase the problem complexity by integrating excessive constraints directly into the optimization process. MPC is an excellent method to handle various constraints, but this approach is often associated with high online computational complexity and time-consuming iterations. To overcome these challenges, we adopt the saturation function to constrain the control input in this study. The saturation function offers a more robust and flexible approach to enforce control input constraints, effectively avoiding the issues posed by the previous methods.

## SADP algorithm

SADP is an advanced methodology to identify an optimal control policy that optimizes the performance function (2) under bounded disturbance and complies with safety constraints (9). At its core, SADP integrates two processes: PEV process that approximates the value of each state-action-disturbance point, i.e., the performance function (2), and PIM process that iteratively refines the policy to minimize this performance function. Uniquely, SADP transforms the traditional PIM into a constrained optimization task with a far-sighted safety function (9) to guarantee execution safety. In addition to the processes described above, game theory is utilized in solving the optimal control policy under disturbances. The disturbance is treated as a competing ‘gamer’ against the optimal policy, aiming to maximize the performance function. However, game theory is primarily used to model the worst-case disturbance scenarios and optimize control responses, rather than directly addressing safety risks.

In this section, an iterative off-policy SADP scheme built upon this PEV and PIM structure (PI manner) is proposed to guide the policy towards a satisfactorily optimal solution. Notably, the iteration rule of the proposed scheme is structured PEV as inner loops and PI as outer loops. For clarity, the inner loop iteration is denoted by $j$, while the outer loop is represented by $i$. Both $i$ and $j$ start from zero and extend to infinity. The way of combining PEV and PI in our ADP framework leverages the strengths of both processes—accurate performance function estimation and effective policy improvement. Also, each improved policy achieved from the completed PEV is still admissible, which is beneficial for extending ADP methods applied in practical scenarios. The detailed iteration rule is outlined as follows.

### Derivation of the off-policy SADP

In regard to off-policy training, various behavior policies are employed for data generation, enhancing data exploitation efficiency. With this approach, the performance function in off-policy and multi-step form can be represented as

10 $$V\left({x}_{t},a,d\right)=\sum_{l=t}^{t+{N}_{p}-1}{\gamma}^{l-t}U\left({x}_{l},a,d\right)+\sum_{l=t+{N}_{p}}^{\infty}{\gamma}^{l-t}U\left({x}_{l},{u}_{l},{\omega}_{l}\right)\,=\,\sum_{l=t}^{t+{N}_{p}-1}U\left({x}_{l},a,d\right)+{\gamma}^{{N}_{p}}{\mathbb{V}}\left({x}_{t+{N}_{p}}\right),$$

where $a\in {\mathcal{U}}_{x}$ and $d\in {\Psi}_{\omega}$. ${\mathcal{U}}_{x}$ represents the safe control input set. In addition, $V\left({x}_{t},a,d\right)$ measures the value of the state ${x}_{t}$ with the behavior policy $a$ and disturbance policy $d$. The optimal performance function ${V}^{*}\left({x}_{t},a,d\right)$ can be given as

11 $${V}^{*}\left({x}_{t},a,d\right)=\underset{a}{\text{min}}\underset{d}{\text{max}}\left(\sum_{l=t}^{t+{N}_{p}-1}{\gamma}^{l-t}U\left({x}_{l},a,d\right)+{\gamma}^{{N}_{p}}{V}^{*}\left({x}_{t+{N}_{p}},{u}_{t+{N}_{p}},{\omega}_{t+{N}_{p}}\right)\right).$$

Based on it, the optimal control and disturbance policy solved via the off-policy manner are given by

12 $${u}_{t}^{*}=\text{arg}\underset{a}{\text{min}}\left({V}^{*}\left({x}_{t},a,d\right)\right){\text{s.t.}} {J}_{c}\left({x}_{c,t}^{{N}_{c}}\right)\le {d}_{c}.$$

13 $${\omega}_{t}^{*}=\text{arg}\underset{d}{\text{max}}\left({V}^{*}\left({x}_{t},a,d\right)\right).$$

To obtain ${u}_{t}^{*}$, the performance function, control policy, and disturbance policy are updated by iterations.

Based on the given initial admissible control policy ${u}_{0,t}\in {\mathcal{U}}_{x}$ and disturbance policy ${\omega}_{0,t}\in {\Psi}_{\omega}$, for $i=\text{1,2},3,\dots,$ the iterative performance function

14 $${V}_{i,j+1}\left({x}_{t},a,d\right)=\sum_{l=t}^{t+{N}_{p}-1}{\gamma}^{l-t}U\left({x}_{l},a,d\right)+{\gamma}^{{N}_{p}}{V}_{i,j}\left({x}_{t+{N}_{p}},{u}_{i,t+{N}_{p}},{\omega}_{i,j,t+{N}_{p}}\right),$$

and the iterative disturbance policy is

15 $${\omega}_{i,j+1,t}=\text{arg}\underset{d}{\text{max}}{V}_{i,j+1}\left({x}_{t},{u}_{i,t},d\right).$$

Then, the iterative control policy can be denoted as

16 $${u}_{i+1,t}=\text{arg}\underset{a}{\text{min}}\left({V}_{i}\left({x}_{t},a,{\omega}_{i,t}\right)\right) {\text{s.t.}}: {J}_{c}\left({x}_{c,t}^{{N}_{c}}\right)\le {d}_{c}.$$

Please note that ${\omega}_{i,t}=$ ${\omega}_{i,\infty, t}$ and ${V}_{i,t}=$ ${V}_{i,\infty, t}$ as $j\to \infty$. Notably, the update of disturbance policy alternates with that of performance function. After both the performance function and disturbance policy converge, the PIM is then implemented.

In this study, the original PIM process is converted into solving the constrained optimization problem described in (16). However, it is almost impossible to obtain the analytical solution directly due to the strong nonlinear property of the objective function and constraints. Motivated by [41], the linearization technique is used to deal with this challenge by linearizing the performance function and state constraints at the corresponding control policy. Based on this, the iterative objective function can be approximated by

17 $${V}_{i}\left({x}_{t},{u}_{t},{\omega}_{i,t}\right)={V}_{i}\left({x}_{t},{u}_{t},{\omega}_{i,t}\right) \vert_{{u}_{t}={u}_{i,t}}+{\left({\nabla}_{{u}_{t}}{V}_{i}\left({x}_{t},{u}_{t},{\omega}_{i,t}\right)\vert_{{u}_{t}={u}_{i,t}}\right)}^{T}\left({u}_{t}-{u}_{i,t}\right)+{R}_{1}^{V}\left({u}_{t}\right),$$

where ${R}_{1}^{V}\left({u}_{t}\right)$ represents the residual error. Then, by substituting (17) into the first line of (16), it becomes evident that the constant term ${V}_{i}\left({x}_{t},{u}_{t},{\omega}_{i,t}\right)\vert_{{u}_{t}={u}_{i,t}}$ in (17) does not affect the optimization process, and the residual error ${R}_{1}^{V}\left({u}_{t}\right)$ can generally be ignored. Thus, the new optimization objective can be expressed as ${u}_{i+1,t}=\text{arg}\underset{{u}_{t}}{\text{min}}{\left({\nabla}_{{u}_{t}}{V}_{i}\left({x}_{t},{u}_{t},{\omega}_{i,t}\right)\vert_{{u}_{t}={u}_{i,t}}\right)}^{T}\left({u}_{t}-{u}_{i,t}\right)$. It is worth noting that the target control policy is denoted as $a$ in (16), but the target control policy in Taylor’s expansion is denoted as ${u}_{t}$ in (17). In (16), $a$ represents the control policy sought to minimize the $i$-th iterative performance function ${V}_{i}\left(\bullet \right)$. However, in (17), for a specific iteration $i$, ${u}_{t}$ effectively serves more like a parameter within the function ${V}_{i}\left(\bullet \right)$.

Furthermore, the approximated safe function can be presented by

18 $${J}_{c}\left({x}_{c,t}^{{N}_{c}}\right)={J}_{c}\left({x}_{c,t}^{{N}_{c}}\right)\vert_{{u}_{t}={u}_{i,t}}+{\left({\nabla}_{{u}_{t}}{J}_{c}\left({x}_{c,t}^{{N}_{c}}\right)\vert_{{u}_{t}={u}_{i,t}}\right)}^{T}\left({u}_{t}-{u}_{i,t}\right)+{R}_{1}^{c}\left({u}_{t}\right),$$

where ${R}_{1}^{c}\left({u}_{t}\right)$ is the residual error. A series of predicted states are derived from initial state ${x}_{c,t}$ with current control policy ${u}_{i,t}$. For the approximation of safe function ${J}_{c}\left({x}_{c,t}^{{N}_{c}}\right)$, the residual error ${R}_{1}^{c}\left({u}_{t}\right)$ can generally be ignored. After this, combining (8) and (18), the state constraint, i.e., the second line of (16), can be expressed as ${J}_{c}\left({x}_{c,t}^{{N}_{c}}\right)\vert_{{u}_{t}={u}_{i,t}}+{\left({\nabla}_{{u}_{t}}{J}_{c}\left({x}_{c,t}^{{N}_{c}}\right)\vert_{{u}_{t}={u}_{i,t}}\right)}^{T}\left({u}_{t}-{u}_{i,t}\right)\le {d}_{c}$.

To guarantee that the approximate schemes (17) and (18) are reasonable, the updated range of iterative control policy should be restricted in a small magnitude scale. To measure the updated range of control policy reasonably, an auxiliary function $D\left({u}_{t},{u}_{i,t}\right)$ is introduced to measure the difference between the new and current control policies, which can be defined as

19 $$D\left({u}_{t},{u}_{i,t}\right)\triangleq {\mathbb{E}}_{{x}_{k}\sim {\vartheta}_{x}}\left[{\Vert {u}_{t}-{u}_{i,t}\Vert}_{2}^{2}\right],$$

where ${\mathbb{E}}_{{x}_{t}\sim {\vartheta}_{x}}$ represents the expectation with regard to the state distribution ${\vartheta}_{x}$ on $\Omega$.

$D\left({u}_{t},{u}_{i,t}\right)$ can also be approximated by linearization:

20 $$ D\left( {u_{t}, u_{i,t}} \right) = D\left( {u_{t}, u_{i,t}} \right)\vert_{{u_{t} = u_{i,t}}} + \left( {\nabla_{{u_{t}}} D\left( {u_{t}, u_{i,t}} \right)\vert_{{u_{t} = u_{i,t}}}} \right)^{T} \left( {u_{t} - u_{i,t}} \right) + \frac{1}{2}\left( {u_{t} - u_{i,t}} \right)^{T} \left( {\nabla_{{u_{t}}}^{2} D\left( {u_{t}, u_{i,t}} \right)\vert_{{u_{t} = u_{i,t}}}} \right)\left( {u_{t} - u_{i,t}} \right) + R_{1}^{D} \left( {u_{t}} \right).$$

The restricted range of $D\left({u}_{t},{u}_{i,t}\right)$ can be denoted as $\delta$. In (20), the constant term $D\left({u}_{t},{u}_{i,t}\right)\vert_{{u}_{t}={u}_{i,t}}$ does not affect the optimization process, and the boundary $\delta$ takes into account the utility of the constant term; thus, it can be removed. For the first-order term, since ${u}_{t}$ is intended to approach the old control policy ${u}_{i,t}$ and the process of searching for a more optimal control policy generally starts from the old policy ${u}_{i,t}$, the first-order term ${\left({\nabla}_{{u}_{t}}D\left({u}_{t},{u}_{i,t}\right)\vert_{{u}_{t}={u}_{i,t}}\right)}^{T}\left({u}_{t}-{u}_{i,t}\right)$ is typically close to zero [39, 41]. Due to this proximity, the first-order term can be ignored. Also, the residual error ${R}_{1}^{D}\left({u}_{t}\right)$ can generally be ignored. Consequently, only the second-order term remains. Therefore, the constraint of restricting the difference between the new and current control policies can be expressed as $\frac{1}{2}{\left({u}_{t}-{u}_{i,t}\right)}^{T}\left({\nabla}_{{u}_{t}}^{2}D\left({u}_{t},{u}_{i,t}\right)\vert_{{u}_{t}={u}_{i,t}}\right)\left({u}_{t}-{u}_{i,t}\right)\le \delta$.

Then, combining (16), (17), (18), and (20), the constrained PIM step can be expressed as follow.

21 $$ \begin{aligned}& u_{i + 1,t} = \arg \mathop {\min}\limits_{{u_{t}}} \left( {\nabla_{{u_{t}}} V_{i} \left( {x_{t}, u_{t}, \omega_{i,t}} \right)\vert_{{u_{t} = u_{i,t}}}} \right)^{T} \left( {u_{t} - u_{i,t}} \right) \hfill \\& {\text{s.t.}}:J_{c} \left( {x_{c,t}^{{N_{c}}}} \right)\vert_{{u_{t} = u_{i,t}}} + \left( {\nabla_{{u_{t}}} J_{c} \left( {x_{c,t}^{{N_{c}}}} \right)\vert_{{u_{t} = u_{i,t}}}} \right)^{T} \left( {u_{t} - u_{i,t}} \right) \le d_{c} \hfill \\& \frac{1}{2} ({u_t} -{u_{i,t}})^{T} (\nabla^{2}_{u_t}D (u_t, u _{i,t})|u_t = u_{i,t} ) (u_{t}-u_{i,t}) \leq \delta. \end{aligned}$$

#### Remark 2:

The introduction of (19) and the last line of (21) are specifically designed to mitigate the adverse effects of approximation errors introduced by using the Taylor expansion technique. The chosen method, which retains the second-order term while ignoring the first-order term of (20), represents one of several valid approaches. The retention of the second-order term of (20) is particularly advantageous as it aligns with the natural gradient method, a strategy inspired by previous studies [39, 41]. It is important to note that retaining both first-order and second-order terms or retaining only the first-order term while ignoring the second-order term of (20) is also a feasible option.

#### Remark 3:

The proposed SADP method ensures safety through both control input and state constraints, with a focus on state constraints. A far-sighted safety function integrates constraints into a constrained optimization process, ensuring that the control policy is updated in a direction that maintains safety while optimizing performance. Unlike CBFs, which require precise models and are difficult to design for multiple constraints, our approach can remain effective in model-free settings. It also avoids the structural disruptions caused by penalty-based methods. However, constrained optimization may lead to infeasible solutions or increased computational costs. To address this, an additional constraint (19) to limit approximation errors and employ techniques such as batch-size optimization and parameter tuning to improve efficiency are introduced in this study.

### Convergence of multi-step off-policy PI SADP

Based on above designs, the convergence of proposed method is developed in this section to show that the performance function ${V}_{i,j}$, the control policy ${u}_{i}$, and the disturbance policy ${\omega}_{i,j}$ can converge to the optimal value as iteration $i,j\to \infty$.

#### Lemma 1:

For fixed control policy ${u}_{i,t}$ where $i=\text{0,1},\dots$, assume the iterative performance function and disturbance policy to be achieved by (14) and (15). Then, for $\forall {x}_{t}\in {\mathbb{R}}^{m}$, the iterative performance function ${V}_{i,j}\left({x}_{t},a,d\right)$ monotonously converge to ${V}_{i,\infty}\left({x}_{t},a,d\right)={V}_{i}\left({x}_{t},a,d\right)$ as $j\to \infty$.

#### Proof:

For $$j=\text{0,1},2,\dots,$$ $${V}_{i,j+1}\left({x}_{t},a,d\right)$$ satisfies.

22 $${V}_{i,j+1}\left({x}_{t},a,d\right)=\sum_{l=t}^{t+{N}_{p}-1}{\gamma}^{l-t}U\left({x}_{l},a,d\right)+{\gamma}^{{N}_{p}}{\mathbb{V}}_{i,j+1}\left({x}_{t+{N}_{p}}\right)=\sum_{l=t}^{t+{N}_{p}-1}{\gamma}^{l-t}U\left({x}_{l},a,d\right)+{\gamma}^{{N}_{p}}\left(U\left({x}_{t+{N}_{p}},{u}_{i,t+{N}_{p}},{\omega}_{i,j+1,t+{N}_{p}}\right)+\gamma {\mathbb{V}}_{i,j+1}\left({x}_{t+{N}_{p}+1}\right)\right).$$

The function $U\left({x}_{t+{N}_{p}},{u}_{i,t+{N}_{p}},{\omega}_{i,j+1,t+{N}_{p}}\right)$ can be written as

23 $$U\left({x}_{t+{N}_{p}},{u}_{i,t+{N}_{p}},{\omega}_{i,j+1,t+{N}_{p}}\right)={V}_{i,j}\left({x}_{t+{N}_{p}},{u}_{i,t+{N}_{p}},{\omega}_{i,j+1,t+{N}_{p}}\right)-\gamma {\mathbb{V}}_{i,j}\left({x}_{t+{N}_{p}+1}\right).$$

Substituting (23) into (22), we have

24 $${V}_{i,j+1}\left({x}_{t},a,d\right)=\sum_{l=t}^{t+{N}_{p}-1}{\gamma}^{l-t}U\left({x}_{l},a,d\right)+{\gamma}^{{N}_{p}}{V}_{i,j}\left({x}_{t+{N}_{p}},{u}_{i,t+{N}_{p}},{\omega}_{i,j+1,t+{N}_{p}}\right)+{\gamma}^{{N}_{p}+1}{\mathbb{V}}_{i,j+1}\left({x}_{t+{N}_{p}+1}\right)-{\gamma}^{{N}_{p}+1}{\mathbb{V}}_{i,j}\left({x}_{t+{N}_{p}+1}\right).$$

Then, based on (15), we can obtain

25 $${V}_{i,j}\left({x}_{t+{N}_{p}},{u}_{i,t+{N}_{p}},{\omega}_{i,j+1,t+{N}_{p}}\right)=\underset{\omega}{\text{max}}{V}_{i,j}\left({x}_{t+{N}_{p}},{u}_{i,t+{N}_{p}},\omega \right)\vert_{\omega ={\omega}_{i,j}}\ge {V}_{i,j}\left({x}_{t+{N}_{p}},{u}_{i,t+{N}_{p}},{\omega}_{i,j,t+{N}_{p}}\right).$$

In fact, the control policy is always deterministic. Thus, the following equation can be obtained

26 $${V}_{i,j}\left({x}_{t+{N}_{p}},{u}_{i,t+{N}_{p}},{\omega}_{i,j,t+{N}_{p}}\right)={\mathbb{V}}_{i,j}\left({x}_{t+{N}_{p}}\right).$$

Subsequently, based on (25) and (26), (24) can be rewritten as

27 $${V}_{i,j+1}\left({x}_{t},a,d\right) \ge \sum_{l=t}^{t+{N}_{p}-1}{\gamma}^{l-t}U\left({x}_{l},a,d\right)+{\gamma}^{{N}_{p}}{\mathbb{V}}_{i,j}\left({x}_{t+{N}_{p}}\right)+{\gamma}^{{N}_{p}+1}{\mathbb{V}}_{i,j+1}\left({x}_{t+{N}_{p}+1}\right)-{\gamma}^{{N}_{p}+1}{\mathbb{V}}_{i,j}\left({x}_{t+{N}_{p}+1}\right)=\sum_{l=t}^{t+{N}_{p}-1}{\gamma}^{l-t}U\left({x}_{l},a,d\right)+{\gamma}^{{N}_{p}}U\left({x}_{t+{N}_{p}},{u}_{i,t+{N}_{p}},{\omega}_{i,j,t+{N}_{p}}\right)+{\gamma}^{{N}_{p}+1}{\mathbb{V}}_{i,j+1}\left({x}_{t+{N}_{p}+1}\right).$$

Then we can get

28 $${V}_{i,j+1}\left({x}_{t},a,d\right)\ge \sum_{l=t}^{t+{N}_{p}-1}{\gamma}^{l-t}U\left({x}_{l},a,d\right)+{\gamma}^{{N}_{p}}U\left({x}_{t+{N}_{p}},{u}_{i,t+{N}_{p}},{\omega}_{i,j,t+{N}_{p}}\right)+{\gamma}^{{N}_{p}+1}\left(U\left({x}_{t+{N}_{p}+1},{u}_{i,t+{N}_{p}+1},{\omega}_{i,j,t+{N}_{p}+1}\right)+\gamma {\mathbb{V}}_{i,j+1}\left({x}_{t+{N}_{p}+2}\right)\right)\ge \sum_{l=t}^{t+{N}_{p}-1}{\gamma}^{l-t}U\left({x}_{l},a,d\right)+{\gamma}^{{N}_{p}}\sum_{l=t+{N}_{p}}^{\infty}{\gamma}^{l-\left(t+{N}_{p}\right)}U\left({x}_{l},{u}_{i,l},{\omega}_{i,j,l}\right)=\sum_{l=t}^{t+{N}_{p}-1}{\gamma}^{l-t}U\left({x}_{l},a,d\right)+{\gamma}^{{N}_{p}}{\mathbb{V}}_{i,j}\left({x}_{t+{N}_{p}}\right)={V}_{i,j}({x}_{t},a,d).$$

So, we have ${V}_{i,j+1}\left({x}_{t},a,d\right)\ge {V}_{i,j}\left({x}_{t},a,d\right)$.

After this, we rewrite the iterative performance function ${V}_{i,j+1}\left({x}_{t},a,d\right)$ as

29 $${V}_{i,j+1}\left({x}_{t},a,d\right)=\sum_{l=t}^{t+{N}_{p}-1}{\gamma}^{l-t}U\left({x}_{l},a,d\right)+{\gamma}^{{N}_{p}}{\mathbb{V}}_{i,j+1}\left({x}_{t+{N}_{p}}\right)=\sum_{l=t}^{t+{N}_{p}-1}{\gamma}^{l-t}U\left({x}_{l},a,d\right) +{\gamma}^{{N}_{p}}{V}_{i,\infty}\left({x}_{t+{N}_{p}},{u}_{i,t+{N}_{p}},{\omega}_{i,j+1,t+{N}_{p}}\right) \quad +{\gamma}^{{N}_{p}}\left[\gamma {\mathbb{V}}_{i,j+1}\left({x}_{t+{N}_{p}+1}\right)-\gamma {\mathbb{V}}_{i,\infty}\left({x}_{t+{N}_{p}+1}\right)\right].$$

Based on (15), we can get

30 $$\begin{aligned}& {V}_{i,\infty}\left({x}_{t+{N}_{p}},{u}_{i,t+{N}_{p}},{\omega}_{i,j+1,t+{N}_{p}}\right)\le  \underset{\omega}{\text{max}}{V}_{i,\infty}\left({x}_{t+{N}_{p}},{u}_{i,t+{N}_{p}},\omega \right) \\& ={V}_{i,\infty}\left({x}_{t+{N}_{p}},{u}_{i,t+{N}_{p}},{\omega}_{i,\infty}\right)={\mathbb{V}}_{i,\infty}\left({x}_{t+{N}_{p}}\right). \end{aligned}$$

By combining (29) and (30), we can obtain

31 $${V}_{i,j+1}\left({x}_{t},a,d\right)\le \sum_{l=t}^{t+{N}_{p}-1}{\gamma}^{l-t}U\left({x}_{l},a,d\right)+{\gamma}^{{N}_{p}}{\mathbb{V}}_{i,\infty}\left({x}_{t+{N}_{p}}\right)+{\gamma}^{{N}_{p}}\left[\gamma {\mathbb{V}}_{i,j+1}\left({x}_{t+{N}_{p}+1}\right)-\gamma {\mathbb{V}}_{i,\infty}\left({x}_{t+{N}_{p}+1}\right)\right]=\sum_{l=t}^{t+{N}_{p}-1}{\gamma}^{l-t}U\left({x}_{l},a,d\right)+{\gamma}^{{N}_{p}}U\left({x}_{t+{N}_{p}},{u}_{i,t+{N}_{p}},{\omega}_{i,\infty, t+{N}_{p}}\right)+{\gamma}^{{N}_{p}+1}U\left({x}_{t+{N}_{p}+1},{u}_{i,t+{N}_{p}+1},{\omega}_{i,\infty, t+{N}_{p}+1}\right)+{\gamma}^{{N}_{p}+2}{\mathbb{V}}_{i,\infty}\left({x}_{t+{N}_{p}+2}\right)=\sum_{l=t}^{t+{N}_{p}-1}{\gamma}^{l-t}U\left({x}_{l},a,d\right)+{\gamma}^{{N}_{p}}{\mathbb{V}}_{t+{N}_{p}}={V}_{i,\infty}\left({x}_{t},a,d\right).$$

Therefore, as $j$ goes to infinity, ${V}_{i,j}\left({x}_{t},{u}_{i,t},{\omega}_{i,j,t}\right)$ will monotonously converge to ${V}_{i,\infty}\left({x}_{t},{u}_{i,t},{\omega}_{i,\infty, t}\right)={V}_{i}\left({x}_{t},{u}_{i,t},{\omega}_{i,t}\right)$.

#### Lemma 2:

Assume that the constrained optimalization problem (21) is solvable, and the iteration rules of ${V}_{i,j}\left({x}_{t},a,d\right)$, ${\omega}_{i,j,t}$, and ${u}_{i,t}$ follow (14), (15), and (21) with ${u}_{i,t}\in {\Psi}_{u}$. Then, for all ${x}_{t}\in\Omega$, the iterative performance function satisfies the monotonic non-increasing property, i.e.,${V}_{i,\infty}\left({x}_{t},a,d\right)\ge {V}_{i+1,\infty}\left({x}_{t},a,d\right)\ge {V}_{\infty, \infty}\left({x}_{t},a,d\right)$.

#### Proof:

Similar to (22)–(26), one has

32 $${V}_{i+1,\infty}\left({x}_{t},a,d\right) =\sum_{l=t}^{t+{N}_{p}-1}{\gamma}^{l-t}U\left({x}_{l},a,d\right)+{\gamma}^{{N}_{p}}{\mathbb{V}}_{i+1,\infty}\left({x}_{t+{N}_{p}}\right)=\sum_{l=t}^{t+{N}_{p}-1}{\gamma}^{l-t}U\left({x}_{l},a,d\right)+{\gamma}^{{N}_{p}}\left(U\left({x}_{t+{N}_{p}},{u}_{i+1,t+{N}_{p}},{\omega}_{i+1,\infty, t+{N}_{p}}\right)+\gamma {\mathbb{V}}_{i,\infty}\left({x}_{t+{N}_{p}+1}\right)+\gamma {\mathbb{V}}_{i+1,\infty}\left({x}_{t+{N}_{p}+1}\right)-\gamma {\mathbb{V}}_{i,\infty}\left({x}_{t+{N}_{p}+1}\right)\right)=\sum_{l=t}^{t+{N}_{p}-1}{\gamma}^{l-t}U\left({x}_{l},a,d\right)+{\gamma}^{{N}_{p}}\left({V}_{i,\infty}({x}_{t+{N}_{p}},{u}_{i+1,t+{N}_{p}},{\omega}_{i+1,\infty, t+{N}_{p}})+\gamma {\mathbb{V}}_{i+1,\infty}\left({x}_{t+{N}_{p}+1}\right)-\gamma {\mathbb{V}}_{i,\infty}\left({x}_{t+{N}_{p}+1}\right)\right).$$

According to (15) and (21), one has

33 $${V}_{i,\infty}\left({x}_{t+{N}_{p}},{u}_{i+1,t+{N}_{p}},{\omega}_{i+1,\infty, t+{N}_{p}}\right)\le {V}_{i,\infty}\left({x}_{t+{N}_{p}},{u}_{i+1,t+{N}_{p}},{\omega}_{i,\infty, t+{N}_{p}}\right).$$

Subsequently, Eq. (32) can be rewritten as

34 $${V}_{i+1,\infty}\left({x}_{t},a,d\right)\le {V}_{i,\infty}\left({x}_{t},a,d\right).$$

As $i\to \infty$, we can also obtain

35 $${V}_{i,\infty}\left({x}_{t},a,d\right)\ge {V}_{i+1,\infty}\left({x}_{t},a,d\right)\ge {V}_{\infty, \infty}\left({x}_{t},a,d\right).$$

#### Theorem 1:

For $i,j=\text{0,1},\dots, \infty,$ let sequences ${V}_{i,j}\left({x}_{t},a,d\right)$, ${\omega}_{i,j,t}$, and ${u}_{i,t}$ be iterated according to (14), (15), (21) with ${u}_{i,t}\in {\Psi}_{u}$. Then, one has ${V}_{i,j}\left({x}_{t},a,d\right)\to {V}_{\infty, \infty}\left({x}_{t},a,d\right)$, and ${u}_{i,t}$ and ${\omega}_{i,j,t}$ converge to ${u}_{t}^{*}$ and ${\omega}_{t}^{*}$, respectively.

#### Proof:

The value function ${\mathbb{V}}_{\infty, \infty}({x}_{t})$ can be denoted as.

36 $${\mathbb{V}}_{\infty, \infty}\left({x}_{t}\right)={V}_{\infty, \infty}\left({x}_{t},{u}_{\infty, t},{\omega}_{\infty, \infty, t}\right)=\underset{u}{\text{min}}\underset{\omega}{\text{max}}{V}_{\infty, \infty}\left({x}_{t},u,\omega \right)=\underset{u}{\text{min}}\underset{\omega}{\text{max}}\left(\sum_{l=t}^{t+{N}_{p}-1}{\gamma}^{l-t}U\left({x}_{l},u,\omega \right)+{\gamma}^{{N}_{p}}{\mathbb{V}}_{\infty, \infty}\left({x}_{t+{N}_{p}}\right)\right)={\mathbb{V}}^{*}\left({x}_{t}\right),$$

where the implementation of $\underset{u}{\text{min}}(\bullet )$ should be subject to constraints. Based on (10) and (11), we have

37 $${V}_{\infty, \infty}\left({x}_{t},a,d\right)=\sum_{l=t}^{t+{N}_{p}-1}{\gamma}^{l-t}U\left({x}_{l},a,d\right)+{\gamma}^{{N}_{p}}{\mathbb{V}}_{\infty, \infty}\left({x}_{t+{N}_{p}}\right)=\sum_{l=t}^{t+{N}_{p}-1}{\gamma}^{l-t}U\left({x}_{l},a,d\right)+{\gamma}^{{N}_{p}}{\mathbb{V}}^{*}\left({x}_{t+{N}_{p}}\right)={V}^{*}\left({x}_{t},a,d\right).$$

We can conclude that $<{u}_{\infty},{\omega}_{\infty, \infty}>$ is the saddle point. Therefore, ${u}_{\infty}={u}^{*}$, ${\omega}_{\infty, \infty}={\omega}^{*}$.

#### Remark 4:

Regarding the stability of closed-loop control system, it can be analyzed through the lens of optimality. A controller resulting from a typical adaptive critic technique assures stability since it is essentially an optimal controller. Optimal control guarantees stability with the nonexistence of conjugate points, ensuring that the control objective moves towards the only equilibrium point based on the optimal solution [42]. Furthermore, according to the boundedness of initial admissible control law and the monotonicity of iterative performance function, it can be deduced that the optimal performance function remains finite. This critical property implies that the optimal control law can effectively stabilize the system, as an infinite performance function indicates an inability to achieve stability.

## Implementation of SADP

In the previous section, the multi-step off-policy SADP scheme considering disturbances is proposed, and the feasibility of the proposed algorithm has been demonstrated through rigorous analysis. In this section, we delve into the practical implementation process of the proposed algorithm, focusing on the specific implemented method for handing control-input and state constraints. Moreover, we explore the integration of NN techniques with the proposed scheme, enabling the algorithm to efficiently learn and optimize the control policy.

### SADP algorithm with NN

**Fig. 1:**
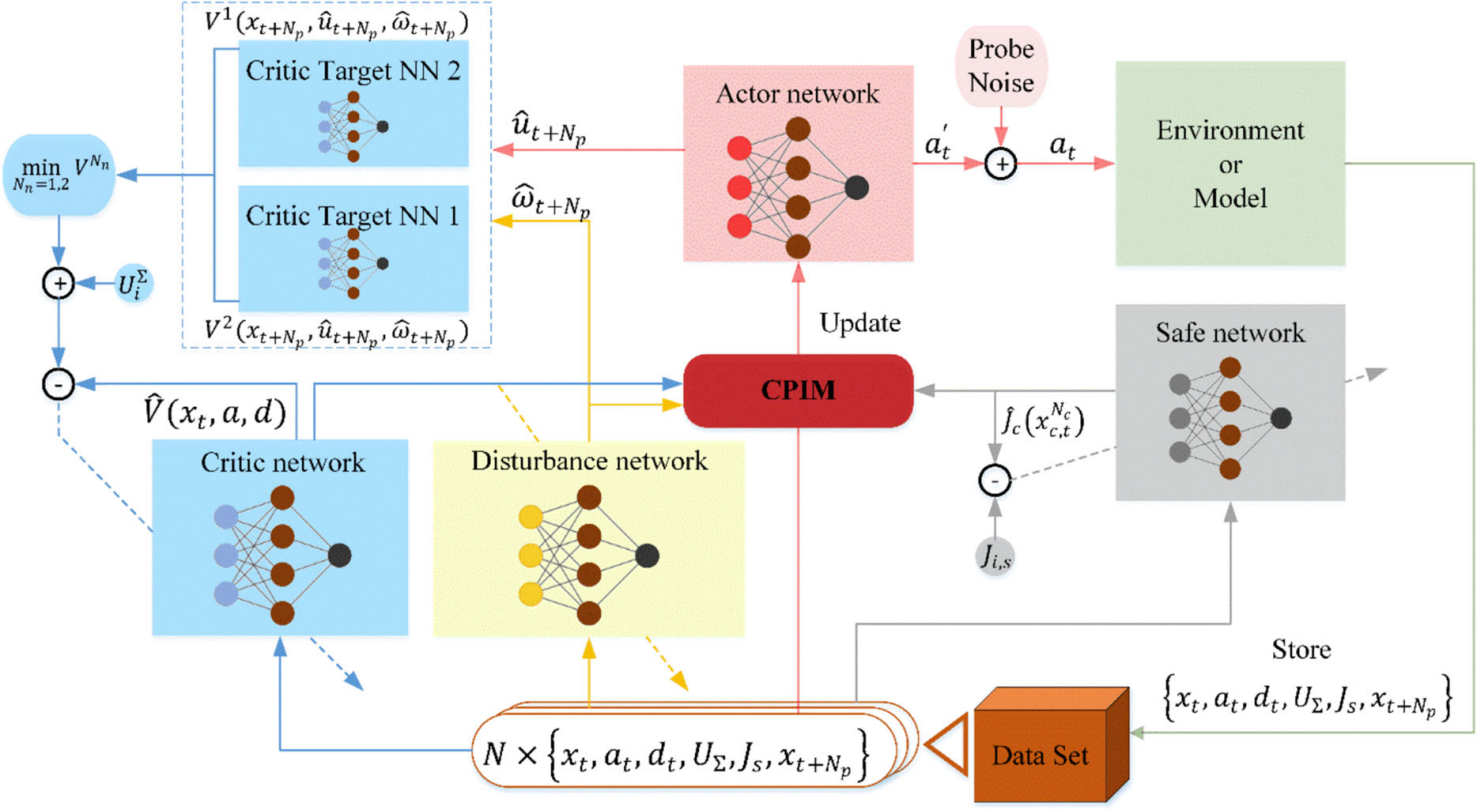
The architecture of the multi-step off-policy SADP considering disturbances algorithm

Based on the above analyses, the main architecture of the proposed multi-step off-policy SADP is shown in Fig. 1, and the main implementation procedure is given in Algorithm 1.

The utilization of NN technique plays a crucial role in implementing the proposed algorithm. Specific to the implementation of the iterative rule (14), (15), and (21) NNs can be employed to approximate ${V}_{i,j}\left({x}_{t},a,d\right)$, ${\omega}_{i,j,t}$, and ${u}_{i,t}$. Also, to effectively achieve the value of safe cost function, a separate NN is introduced to approximate it. In the following subsections, we demonstrate the specific iterative update rule of each NN involved.

**Algorithm 1:**
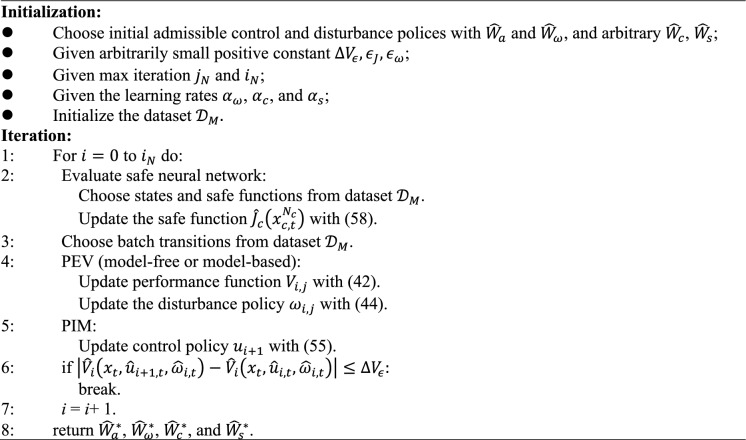
SADP Algorithm with NN

### Implementation of the critic network

A single hidden layer NN is used to evaluate the performance function $\widehat{V}\left({x}_{t},a,d\right)$, which can be expressed as

38 $$ \hat{V}_{{i,j}} \left( {x_{t}, a,d} \right) = \hat{W}_{{i,j}}^{{cT}} \sigma _{c} \left( {Y_{c}^{T} \left( {x_{t}, a,d} \right)} \right),$$

where ${\widehat{W}}_{i,j}^{c}$ represents the weight vector between hidden layer and output layer with PEV inner loop $j$ and PI outer loop $i$, and ${Y}_{c}$ represents the weight matrix between hidden layer and input layer. A nonlinear activation function ${\sigma}_{c}\left(\bullet \right)$ is used typically after the output of hidden layer, which can improve the nonlinear property, thereby advancing the approximation precision of performance function. Moreover, ${\sigma}_{c}\left(\bullet \right)$ is supposed to be bounded, i.e., $\Vert {\sigma}_{c}\left({Y}_{c}^{T}\left({x}_{t},a,d\right)\right)\Vert \le {\sigma}_{cM}$, where ${\sigma}_{cM}$ is a positive constant.

In each PEV step, the backpropagating mechanism is adopted to update the weights of critic NN. The loss function can be represented as

39 $${E}_{i,j}^{c}=\frac{1}{2}{\Vert {e}_{i,j}^{c}\Vert}^{2},$$

where

40 $${e}_{i,j}^{c}={\widehat{V}}_{i,j}\left({x}_{t},a,d\right)-\sum_{l=t}^{t+{N}_{p}-1}{\gamma}^{l-t}U\left({x}_{l},a,d\right)-{\gamma}^{{N}_{p}}{\widehat{V}}_{i,j}\left({x}_{t+{N}_{p}},{\widehat{u}}_{i,t+{N}_{p}},{\widehat{\omega}}_{i,j-1,t+{N}_{p}}\right),$$

41 $${\widehat{V}}_{i,j,t}^{target}\triangleq \sum_{l=t}^{t+{N}_{p}-1}{\gamma}^{l-t}U\left({x}_{l},a,d\right)+{\gamma}^{{N}_{p}}{\widehat{V}}_{i,j}\left({x}_{t+{N}_{p}},{\widehat{u}}_{i,t+{N}_{p}},{\widehat{\omega}}_{i,j-1,t+{N}_{p}}\right).$$

Subsequently, the weight matrix ${\widehat{W}}_{i,j}^{c}$ can be updated as

42 $${\widehat{W}}_{i,j+1}^{c}={\widehat{W}}_{i,j}^{c}+\Delta {\widehat{W}}_{i,j}^{c}={\widehat{W}}_{i,j}^{c}-{\alpha}_{c}\frac{\partial {E}_{i,j}^{c}}{\partial {e}_{i,j}^{c}}\frac{\partial {e}_{i,j}^{c}}{\partial {\widehat{V}}_{i,j}\left({x}_{t},a,d\right)}\frac{\partial {\widehat{V}}_{i,j}\left({x}_{t},a,d\right)}{\partial {\widehat{W}}_{i,j}^{c}}={\widehat{W}}_{i,j}^{c}-{\alpha}_{c}{e}_{i,j}^{c}{\sigma}_{c}\left({Y}_{C}^{T}\left({x}_{t},a,d\right)\right),$$

where $0<{\alpha}_{c}\le 1$ represents the learning rate of the critic network.

### Implementation of the disturbance network

The disturbance network is used to estimate the worst-case disturbance ${\omega}_{t}^{*}$. The iterative disturbance policy ${\widehat{\omega}}_{i,t}$ can be represented as

43 $${\widehat{\omega}}_{i,j,t}={{\widehat{W}}_{i,j}^{\omega}}{T}{\sigma}_{\omega}\left({Y}_{\omega}^{T}\left({x}_{t}\right)\right),$$

where ${\widehat{W}}_{i,j}^{\omega}$ and ${Y}_{\omega}$ represent the weights of the disturbance network. In a similar way, a nonlinear activation function ${\sigma}_{\omega}\left(\bullet \right)$ is implemented in the disturbance network.

The policy gradient technique can be used to update the iterative disturbance. The corresponding weights can be updated by the following equation,

44 $${\widehat{W}}_{i,j+1}^{\omega}={\widehat{W}}_{i,j}^{\omega}+\Delta {\widehat{W}}_{i,j}^{\omega}={\widehat{W}}_{i,j}^{\omega}-{\alpha}_{\omega}\left(-\frac{\partial {\widehat{V}}_{i,j+1}\left({x}_{t},{\widehat{u}}_{i,t},{\widehat{\omega}}_{i,j,t}\right)}{\partial {\widehat{\omega}}_{i,j}}\frac{\partial {\widehat{\omega}}_{i,j}}{\partial {\widehat{W}}_{i,j}^{\omega}}\right)={\widehat{W}}_{i.j}^{\omega}+{\alpha}_{\omega}\left({\sigma}_{\omega}\left({Y}_{\omega}^{T}\left({x}_{t}\right)\right)\bullet {{\widehat{W}}_{i,j+1}^{cT}}  \otimes   {\sigma}_{c}{\prime}\left({Y}_{c}^{T}\left({x}_{t},{\widehat{u}}_{i,t},{\widehat{\omega}}_{i,j,t}\right)\bullet {Y}_{c}^{T}\left(-n:end,:\right)\right)\right),$$

where $\otimes$ represents the elementwise multiplication, and $0<{\alpha}_{\omega}\le 1$ denotes the NN learning rate.

### Implementation of the actor network

The actor network is to approximate the iterative control input ${\widehat{u}}_{i,t}$ following

45 $${\widehat{u}}_{i,t}=\upzeta \left({{\widehat{W}}_{i}^{aT}}{\sigma}_{a}\left({Y}_{a}^{T}\left({x}_{t}\right)\right) \right),$$

where ${\widehat{W}}_{i}^{a}$ and ${Y}_{a}$ represent the weights of the actor network. ${\sigma}_{a}\left(\bullet \right)$ is a nonlinear activation function. To ensure the saturation characteristic of the control policy, another nonlinear activation function $\upzeta \left(\bullet \right)$ is introduced at the output layer of the actor network, and $\left| {\zeta \left( {\hat{W}_{i}^{{aT}} \sigma _{a} \left( {Y_{a}^{T} \left( {x_{t}} \right)} \right)~} \right)} \right| \le \bar{U}$, where $\overline{U}={\left[{\overline{u}}_{1},{\overline{u}}_{2},\dots, {\overline{u}}_{n}\right]}^{T}$.

Due to the fact that the traditional PIM step using the gradient descent method can only handle unconstrained optimization problems, the feasible PIM step in SADP is reconstructed. For simplicity, based on (21), the constrained PIM step can be expressed as:

$$\underset{u}{\text{min}}{g}^{T}\Delta {\widehat{W}}^{a}$$

46 $${\text{s.t.}}: c+{b}^{T}\Delta {\widehat{W}}^{a}\le 0 \frac{1}{2}{\left(\Delta {\widehat{W}}^{a}\right)}^{T}H\left(\Delta {\widehat{W}}^{a}\right)<\delta,$$

where $$\Delta {\widehat{W}}^{a}={\widehat{W}}^{a}-{\widehat{W}}_{i}^{a}$$, and

47 $$g={\sigma}_{a}\left({Y}_{a}^{T}\left({x}_{t}\right)\right)\bullet {{\widehat{W}}^{cT}}  \otimes  {\sigma}_{c}{\prime}\left({Y}_{c}^{T}\left({x}_{t},{\widehat{u}}_{i,t},{\widehat{\omega}}_{i,t}\right)\right)\bullet Y_{c,\left(m:m+n,:\right)}^{T}\left({x}_{t},a,d\right),$$

48 $$b={\left({\nabla}_{u}{J}_{c}\left({x}_{c,t}^{{N}_{c}}\right)\vert_{u={u}_{i}}\right)}^{T},$$

49 $$c={J}_{c}\left({x}_{c,t}^{{N}_{c}}\right)\vert_{u={u}_{i}}-{d}_{c},$$

50 $$H={\nabla}_{{u}_{t}}^{2}D\left({u}_{t},{u}_{i,t}\right)\vert_{{u}_{t}={u}_{i,t}}=\frac{{\partial}^{2}D\left({u}_{t},{u}_{i,t}\right)}{\partial {\widehat{W}}_{i,p}^{a}\partial {\widehat{W}}_{i,q}^{a}}\vert_{{u}_{t}={u}_{i,t}},$$

where both ${\widehat{W}}_{i,p}^{a}$ and ${\widehat{W}}_{i,q}^{a}$ represent a specific weight element of actor network at $i$-th iteration. $p$ and $q$ denote specific positions in the weight matrix ${\widehat{W}}^{a}$. Additionally, $c, \delta \in {\mathbb{R}}$, $\delta >0$, and the Fisher matrix $H$ is positive-definite.

For the problem described in (46), the variable to be optimized is $\Delta {\widehat{W}}^{a}$. Therefore, the iterative weight parameters of the actor network can be expressed as

51 $${\widehat{W}}_{i+1}^{a}={\widehat{W}}_{i}^{a}+\Delta {\widehat{W}}_{i}^{a}.$$

Lagrange multiplier method, as a typically optimalization method in the mathematical community, is adopted to solve (46). First, the Lagrange function is constructed as

52 $$L\left(\Delta {\widehat{W}}^{a},{\lambda}_{1},{\lambda}_{2}\right)={g}^{T}\Delta {\widehat{W}}^{a}+{\lambda}_{1}\left(c+{b}^{T}\Delta {\widehat{W}}^{a}\right)+{\lambda}_{2}\left(\frac{1}{2}{\left(\Delta {\widehat{W}}^{a}\right)}^{T}H\left(\Delta {\widehat{W}}^{a}\right)-\delta \right),$$

where ${\lambda}_{1}$ and ${\lambda}_{2}$ are dual variables. Then, the dual function can be constructed as

53 $$g\left({\lambda}_{1},{\lambda}_{2}\right)=\underset{x\in\Omega}{\text{inf}}\left({g}^{T}\Delta {\widehat{W}}^{a}+{\lambda}_{1}\left(c+{b}^{T}\Delta {\widehat{W}}^{a}\right)+{\lambda}_{2}\left(\frac{1}{2}{\left(\Delta {\widehat{W}}^{a}\right)}^{T}H\left(\Delta {\widehat{W}}^{a}\right)-\delta \right)\right).$$

Following this, the dual problem corresponding original optimization problem (46) can be represented as

54 $$\underset{{\lambda}_{1}\ge 0,{\lambda}_{2}\ge 0}{\text{max}}\underset{\Delta {\widehat{W}}^{a}}{\text{min}}L\left(\Delta {\widehat{W}}^{a},{\lambda}_{1},{\lambda}_{2}\right).$$

Then, the optimal solution $\Delta {\widehat{W}}^{a,*}$ is

55 $$\Delta {\widehat{W}}^{a,*}=-\frac{1}{{\lambda}_{2}^{*}}{H}^{-1}\left(g+{\lambda}_{1}^{*}b\right),$$

where ${\lambda}_{1}^{*}$ and ${\lambda}_{2}^{*}$ are the optimal analytical solutions of dual problem (54).

### Implementation of the constrained safe network

The far-sighted safe function ${J}_{c}\left({x}_{c,t}^{{N}_{c}}\right)$ for the constrained state ${x}_{c,t}$ also can be evaluated by a NN:

56 $${\widehat{J}}_{c}\left({x}_{c,t}^{{N}_{c}}\right)={{\widehat{W}}_{p}^{sT}}{\sigma}_{s}\left({Y}_{s}^{T}\left(\left({x}_{c,t},{\widehat{u}}_{t},{\widehat{\omega}}_{t}\right)\right)\right),$$

where ${\widehat{W}}_{p}^{s}$ and ${Y}_{s}$ denote the weights in the NN structure, and $p$ denotes the number of iterations in the process of approximating the safe function ${J}_{c}\left({x}_{c,t}^{{N}_{c}}\right)$. Moreover, ${\sigma}_{s}(\bullet )$ represents a typical activation.

The training objective of the constrained safe function is $\sum_{l=t}^{{t+N}_{c}-1}{\gamma}_{c}^{l-t}h\left({x}_{c,l}\right)$. Thus, the objective function can be expressed as

57 $${E}_{p}^{s}=\frac{1}{2}{\Vert {e}_{p}^{s}\Vert}^{2},$$

where ${e}_{p}^{s}={\widehat{J}}_{c}\left({x}_{c,t}^{{N}_{c}}\right)-\sum_{l=t}^{{t+N}_{c}-1}{\gamma}_{c}^{l-t}h\left({x}_{c,l}\right)$. Then, based on (57), the weight update law follows

58 $${\widehat{W}}_{p}^{s}={\widehat{W}}_{p}^{s}+\Delta {\widehat{W}}_{p}^{s}={\widehat{W}}_{p}^{s}-{\alpha}_{s}\frac{\partial {E}_{p}^{s}}{\partial {e}_{p}^{s}}\frac{\partial {e}_{p}^{s}}{\partial {\widehat{J}}_{c}\left({x}_{c,t}^{{N}_{c}}\right)}\frac{\partial {\widehat{J}}_{c}\left({x}_{c,t}^{{N}_{c}}\right)}{\partial {\widehat{W}}_{p}^{s}}={\widehat{W}}_{p}^{s}-{\alpha}_{s}{e}_{p}^{s}{\sigma}_{s}\left({Y}_{s}^{T}\left({x}_{t},{\widehat{u}}_{t},{\widehat{\omega}}_{t}\right)\right),$$

where $0<{\alpha}_{s}\le 1$ is the learning rate.

### Performance improvement for SADP with NN

Although the off-policy version offers wider applicability in more general cases, traditional off-policy ADP schemes, in fact, struggle to effectively handle both the PEV process in (14). This is because the target value, i.e., Eq. (41), while updating critic network is not ideal and completely accurate for the iterative performance function based on policy $a$ and disturbance $d$. More specifically, we can observe that the target performance function, i.e., the right side of (14), consists of two part: the accumulation of utility function $\sum_{l=t}^{t+{N}_{p}-1}{\gamma}^{l-t}U\left({x}_{l},a,d\right)$ and terminal performance function ${\gamma}^{{N}_{p}}{\widehat{V}}_{i,j}\left({x}_{t+{N}_{p}},{\widehat{u}}_{i,t+{N}_{p}},{\widehat{\omega}}_{i,j,t+{N}_{p}}\right)$. To evaluated $V\left({x}_{t},a,d\right)$ accurately, a series of control inputs should be identical in the accumulated utility function. However, for the most of multi-step off-policy evaluation methods [43, 44], the control policies executed on consecutive states $({x}_{t},{x}_{t+1},\dots, {x}_{t+{N}_{p}})$ are different due to the consistent PIM step. In addition, the control policy in terminal performance function is a target policy that differs from the behaviour policy in $V\left({x}_{t},a,d\right)$. As a result, a discrepancy may exist between the ideal and target performance function, manifesting as an underestimation for the PEV step.

Based on the above analyses, to effectively address the underestimation in PEV, a multi-step PEV algorithm with interleaved collection and training manner is proposed. Also, this scheme is designed to be adaptable for various practical scenarios, including both model-based and model-free variants. When the model information can be achieved beforehand, multi-step transitions including future states can be derived from the state ${x}_{t}$ using model information. With this approach, any state in $\Omega$ can progress a certain step with the dynamics model, meaning that any state in $\Omega$ can be used for training, which improves both the data utilization and completeness. The model-based PEV implemented in SADP algorithm is presented in Algorithm 2.

**Algorithm 2:**
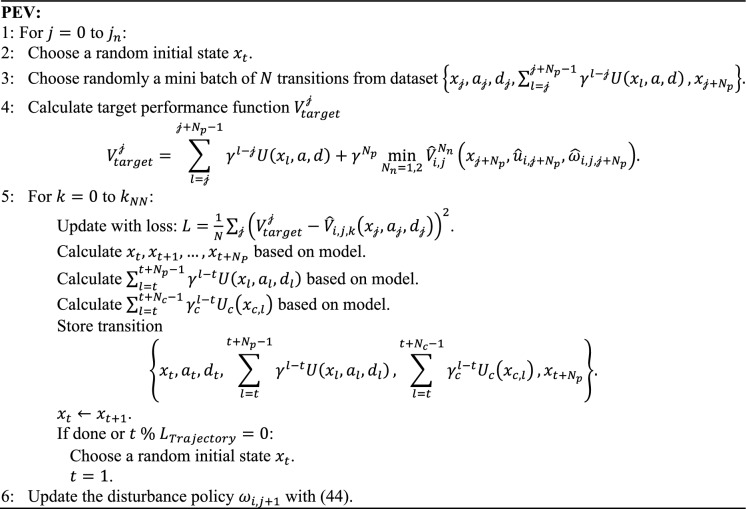
Model-based PEV for SADP

#### Remark 5:

Using the model-based scheme, we can ensure not only the guarantee of data utilization and completeness, but also naturally avoid the drawback of inconsistent control policies within the accumulated utility function. This is because future states from ${x}_{t}$ are derived from the same control policy. In addition, inspired by [45], we introduce the concept of double critic NNs to eliminate the underestimation of the terminal performance function. However, in this study, we eliminate the need to introduce many NNs to achieve underestimation elimination. This is because the iteration method adopted in [45] essentially used a VI method, while our study implements a PI approach. This means that the control policy update occurs after each PEV process, which ensures a complete evaluation of the performance function that is not influenced by the alternating iteration between the performance function and control policy. As a result, better training performance can be achieved by introducing only one additional target critic NN.

Although excellent data utilization and completeness can be obtained with the model-based method, the forward development process is computationally expensive, and this consumption increases exponentially with the complexity of the model. Moreover, it is often challenging to establish an accurate dynamics model in many practical applications [46]. Therefore, proposing a model-free version/variant of SADP is important. The key factor in such a design is how to collect data while evaluating and improving the policy. In [43, 44], we observe that the evaluation and improvement steps occur after each data collection. Such a real-time update method leads to an inaccurate PEV. To mitigate this issue, the series of control inputs within the accumulation of the utility function should maintain consistent policies. In other words, each transition should be collected using the same policy. Based on these analyses, our model-free PEV implemented in SADP algorithm is presented in Algorithm 3.

**Algorithm 3:**
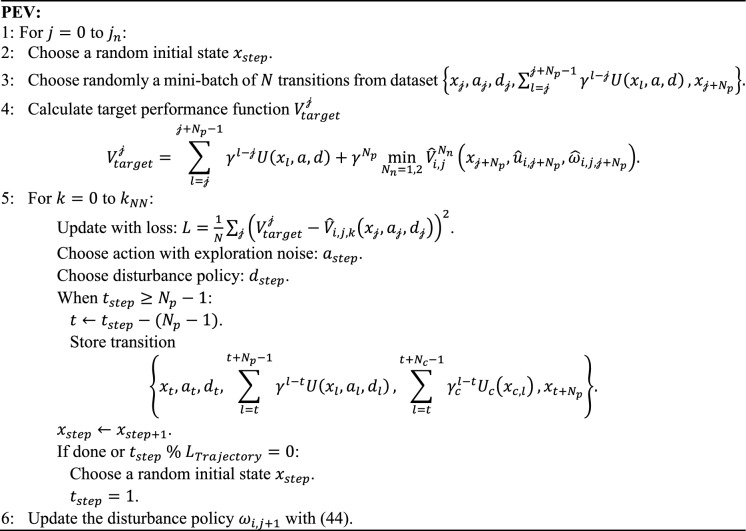
Model-free PEV for SADP

#### Remark 6:

Inspired by [47], a dataset ${\mathcal{D}}_{M}$ is introduced in the model-free version, but the stored content in the dataset ${\mathcal{D}}_{M}$ is different from that in [47]. To be more specific, the transition stored in ${\mathcal{D}}_{M}$ can be represented as $\left\{{x}_{t},{a}_{t},{d}_{t},{\sum}_{p=t}^{t+{N}_{p}-1}{\gamma}^{p-t}{U}_{p},\sum_{l=t}^{{t+N}_{c}-1}{\gamma}_{c}^{l-t}{U}_{c}\left({x}_{c,l}\right),{x}_{t+{N}_{p}}\right\}$, where $M$ denotes the number of samples in the dataset, and the subtotal utility function and subtotal safe function are saved into each transition. Furthermore, as previously mentioned, this study adopts the PI approach. Consequently, the approach of resetting the initial state only when the state exceeds the safe boundary is not suitable. To address this problem, we deliberately set the collected length of the trajectory ${L}_{Trajectory}$. Essentially, when the number of forward steps reaches the upper limit defined by ${L}_{Trajectory}$, the state should also be reset.

#### Remark 7:

From **Algorithm 3**, we can observe that data collection is embedded in the PEV process. This approach ensures that the same behavior policy is always used for data collection, guaranteeing the accurate accumulation of the utility function. Furthermore, similar to the model-based approach, the same concept of double NNs is employed to alleviate the underestimation.

#### Remark 8:

To evaluate the initial control policy under the initial disturbance policy, the dataset initialization should include comprehensive data to enhance the completeness of the approximation. As such, the initial admissible control policy and various random control policies are used to generate transitions. Additionally, the proportion of transitions generated by different control policies can be adjusted.

#### Remark 9:

In the processes of evaluating safe function and PIM, the data utilization is different from that in PEV step. To be more specific, the number of transitions in the PEV step is a small batch size, but the number of transitions in evaluating safe function and PIM step can be more sufficient, or even traverses the whole state-action-disturbance space. This is because in the PEV process, the computation is more sensitive than that in other process. Therefore, if a considerable of transitions are used, it will cause the curse of dimensionality that must be avoided in the PEV process, but for the training process of evaluating safe function, it belongs to supervised learning that can accept more data to training without failure. As for PIM process, it is essentially an optimization problem, i.e., more comprehensive data can be used in PIM.

#### Remark 10:

In the field of safe reinforcement learning, methods to ensure safety often involve modifying the reward structure. For instance, reward shaping [48] is employed to introduce additional costs when an agent approaches or exceeds the boundaries of a safe region, thereby discouraging unsafe actions. Another approach in safe reinforcement learning involves formulating a dual problem that indirectly addresses the original optimization challenge [41, 49], guiding the agent to maintain safety while seeking optimal performance. In this study, the original unconstrained policy optimization is transformed into a Lagrange dual problem. This transformation, coupled with the application of policy iteration starting from an initial admissible control law, ensures asymptotic convergence to an optimal solution while ensuring safety. However, in safe reinforcement learning, particularly when using Lagrange methods for constraint handling, the training process may suffer from instability, and lacks assurance that each policy iteration improves with admissibility. Additionally, the safe ADP community predominantly focuses on control input constraints rather than state constraints [20, 21, 40]. This is because the control input constraints can be guaranteed by constructing a novel utility function, a solution not applicable to state constraints. To navigate this challenge, other researchers have proposed converting the original system into a new model [33], thereby transforming constrained states into unconstrained ones. Although this model transformation technique facilitates the management of constraints, it is an indirect method and may potentially introduce additional bias during the iterative process.

#### Remark 11:

Compared to our previous work [50], which employed on the on-policy framework, the current study introduces an off-policy SADP approach, enabling more efficient data utilization and improved exploration–exploitation balance. To address the underestimation bias inherent in off-policy learning, we propose both model-based and model-free schemes, incorporating an interleaved training mechanism and leveraging prior model knowledge, along with dual critic networks for enhanced stability. Additionally, unlike [50], where the disturbance policy, control policy, and PEV process are updated iteratively in separate steps, the current work integrates the disturbance policy update directly into the PEV process, presenting an effective iterative scheme. Furthermore, we introduce a more intuitive and computationally efficient approach for handling safety constraints by aggregating them into a single inequality involving a far-sighted safety function, simplifying constraint enforcement and reducing computational complexity. These improvements collectively enhance the robustness, efficiency, and safety-awareness of the proposed method.

## Simulation

### Parameter setting

In this section, a simulation case is presented to examine the control performance and safe effectiveness of the proposed approaches that are model-free and model-based multi-step off-policy SADP schemes with the PI method.

First, a pendulum system was adopted to validate the performance [51].

59 $$\left\{\begin{array}{c}\dot{{\theta}_{p}}={\mathcalligra{w}}_{p}\\ J\dot{{\mathcalligra{w}}_{p}}=-Mgl\text{sin}{\theta}_{p}-{f}_{d}\dot{{\theta}_{p}}+{u}_{p}-W{\omega}_{p}\end{array}\right.,$$

where model parameter mass $M=1/3$ and pendulum length $l=2/3$. According to [51], the rotary inertia $J=\frac{4}{3}M{l}^{2}=\frac{16}{81}$ and the frictional factor ${f}_{d}=0.2$. Besides, the system weight of disturbance can be given as $W=1/20$. Normally, the gravitational acceleration can be given by $g=9.8m/{s}^{2}$. Then, the Euler and trapezoidal method is used to discretize the original pendulum system (59) with the discrete time ${\Delta}_{t}=0.2s$. Therefore, the specific discrete-time dynamics model can be represented as

60 $$\left[\begin{array}{c}{x}_{1,t+1}\\ {x}_{2,t+1}\end{array}\right]=\left[\begin{array}{c}0.2{x}_{2,t}+{x}_{1,t}\\ -2\text{sin}{x}_{1,t}+0.8{x}_{2,t}\end{array}\right]+\left[\begin{array}{c}0\\ 1\end{array}\right]{u}_{p,t}-\left[\begin{array}{c}0\\ 0.05\end{array}\right]{\omega}_{p,t},$$

where ${x}_{1}={\theta}_{p}$ and ${x}_{2}={\mathcal{w}}_{p}$. The weight matrices $Q$ and $R$ are set as $Q=\text{diag}\left\{\text{1,1}\right\}$ and $R=\left[0.05\right]$. Also, $\beta =\left[0.05\right]$.

**Fig. 2:**
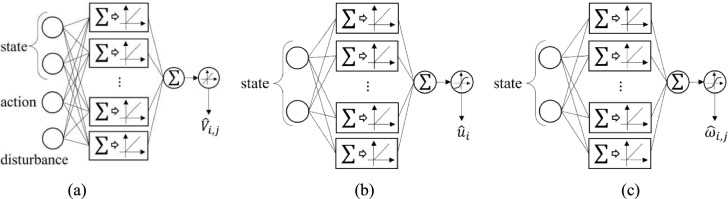
The structures of critic, actor, and disturbance NN. **a** Critic NN. **b** Actor NN. **c** Disturbance NN

In terms of neural network structures, four single-layer NNs are used to approximate various functions: the iterative performance function, the control policy, the disturbance policy and the constrained safe function. Specifically, as shown in Fig. 2, the structures of critic, actor, and disturbance NNs are designed with 4–32-1, 2–32-1, 2–32-1. Notably, the constrained safe NN only undergoes supervised training; hence its structure is not detailed specially. Additionally, regarding the implementation of activation functions, it depends on the specific training model and the functional properties. For the critic and constrained safe NNs, the activation functions for both hidden and output layers are rectified linear unit (ReLU) function. Similarly, the actor and disturbance NNs also employ the ReLU function for the hidden layer. However, to guarantee saturation characteristic of control input and the bounded feature of disturbance, the hyperbolic tangent function is applied for the output layer of actor and disturbance NNs.

To achieve a satisfied balance between convergence speed and stability, the learning rate ${\alpha}_{c}$, ${\alpha}_{\omega}$ and ${\alpha}_{s}$ are both set as $1\times {10}^{-3}$, and the Adam update criteria is adopted for each NNs. In addition, in order to ensure the iterative feasibility of the proposed algorithm, the convergence threshold ${\epsilon}_{J}$ and $\Delta {V}_{\epsilon}$ are both given as ${10}^{-6}$. Furthermore, the capacity of the dataset ${\mathcal{D}}_{M}$ is chosen as ${10}^{4}$ in which initial transitions are collected with various control policies to guarantee the comprehensiveness and completeness of training data. In terms of batch size during PEV process, larger batch sizes can offer faster computation due to parallel processing, but they might also lead to generalization issues or require more memory; hence, the batch size is chosen as ${2}^{10}$ at each iteration step.

To ensure that the pendulum system can be operated within a proper range, the control policy should be limited within $\left|{u}_{p,t}\right|\le 2.0$ according to the saturation characteristics of the actuator. In terms of state constraints, based on the physical limitations of the pendulum system, both system states ${\theta}_{p}$ and ${\mathcal{w}}_{p}$ are bounded by $\left|{x}_{t}\right|\le 1.57\approx {90}^{^\circ}$. Also, considering maximum expected level of disturbance under normal operating conditions, the disturbance is bounded by $\left|{\omega}_{p,t}\right|\le 1.0$. Different initial states can be used to ensure that the control system is versatile and can handle various starting conditions effectively. To this end, a random initial state of the pendulum system is given by ${x}_{0}={\left[0.8,-0.5\right]}^{T}$. A proper number of forward steps is required to adequately represent the system's dynamics, ensuring that the computational process remains efficient without introducing undue complexity or excessive temporal demands. Preliminary tests indicate that setting the forward steps as 15 achieves a satisfactory balance.

### Result analysis

**Fig. 3:**
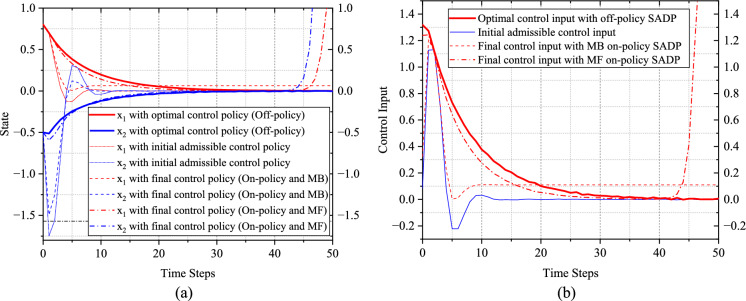
Simulation results of Example 1. **a** State convergence trajectories with on-policy and off-policy. **b** Control policy convergence trajectories with on-policy and off-policy

**Fig. 4:**
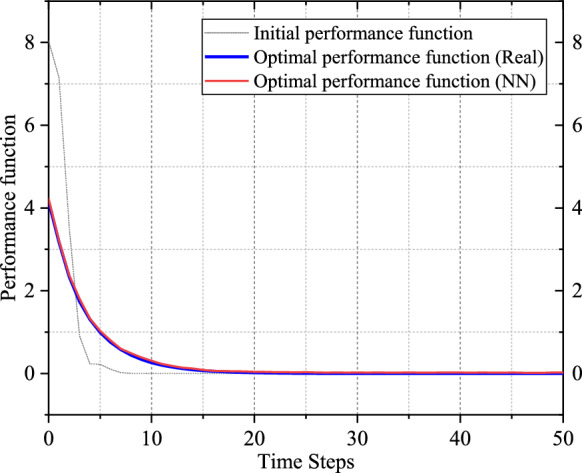
Performance function convergence trajectories with the proposed SADP scheme

The convergence behaviors by implementing the optimal control policy solved by the proposed SADP scheme on (59) are shown in Fig. 3. Notably, all the convergence behaviors generated by various control policies shown in Fig. 3 are response to the system (59) subject to random disturbance. Based on the random disturbance and the optimal control policy in Fig. 3, the control objective demonstrated excellent optimal performance against the existing random disturbance, which shows the effectiveness of the proposed scheme. Also, as shown in Fig. 4, the performance in terms of optimality has been greatly improved compared with using the initial admissible control policy. This precise evaluation forms a crucial foundation of guiding iterative processes towards achieving the reliable and optimal solution. In addition, it is evident that the convergence based on the initial admissible control law is faster than that achieved with optimal control law. This disparity can be attributed to the large magnitude of control input associated with the initial control law, as shown in Fig. 3. It is worth noting that a larger control input could lead to faster convergence due to the immediate impact on system dynamics. However, this expedited convergence comes at the expense of potentially heavier utility function, resulting in a larger accumulated performance function compared to those achieved with the optimal control policy.

Upon closer examination of Fig. 3, by comparing state trajectories executed by initial and optimal control policies, we can find that the state variable ${\mathcal{w}}_{p}$ exceeds the specified range conspicuously with the initial admissible control policy, but after iterations, the final state behaviors can be limited within the state constraint strictly, which validates the safety of the proposed SADP scheme.

Additionally, as shown in Fig. 3, the final convergence behaviors solved by on-policy SADP, developed in [50], are dissatisfactory whether it is model-based or model free schemes. For the model-based version, there exists significant steady state error for the state ${\theta}_{p}$, which is unacceptable during controlling an object. The reason for this phenomenon is that the approximation extent of data distributed in the whole state-action-disturbance space is not complete absolutely. Besides, like mentioned in [16], in order to guarantee the update of NNs, the exploration noise is implemented, which will definitely cause the bias between the optimal and final iterative results ultimately. For the model-free version, the uncompleted evaluation and insufficient exploration cause that the iterative control policy cannot render the system (59) stable. In addition, since there is no dynamics model to provide accurately forward state information, the final iterative results solved by the model-free scheme are more terrible than that in the model-based scheme.

**Fig. 5:**
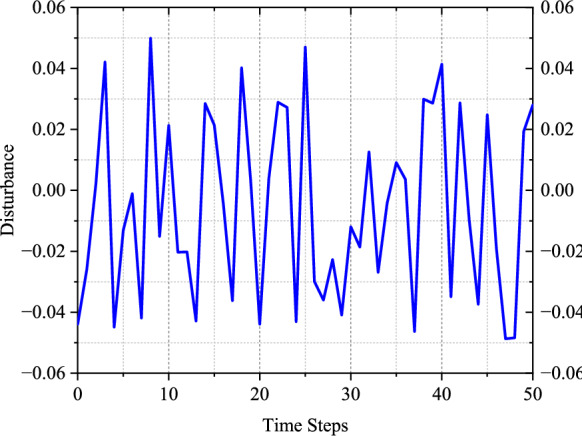
Random disturbance implemented on system

**Fig. 6:**
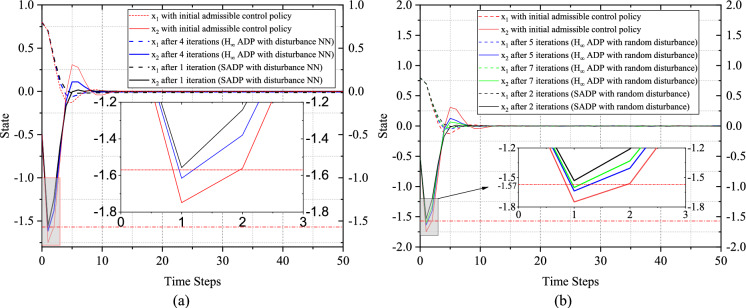
The convergence behavior of state under random disturbances and disturbance NN. **a** Disturbance NN. **b** Random disturbances

To evaluate the robustness of the proposed SADP scheme, we conducted comparative analyses between SADP and traditional ${H}_{\infty}$ ADP [52] under two distinct types of disturbances, i.e., disturbance generated by NN and random disturbance. Because the disturbance existing in the system or environment is stochastic generally, a random disturbance form is considered to more reasonably validate the anti-disturbance capability of the proposed scheme. As shown in Fig. 5, this random disturbance follows a uniform distribution within the range of (-0.05, 0.05). In the analysis of Fig. 6(a), one can see that the traditional ${H}_{\infty}$ ADP scheme cannot guarantee safety within the initial iterations when subjected to the disturbance generated by NN. In contrast, the proposed SADP scheme can render the state behaviors into the safe bound after just the first iteration. This comparison indicates that by addressing the constrained optimization problem under the worst-case disturbance policy, a safe and improved control policy can be achieved immediately, thereby effectively constraining the negative effects that could result in the deviation of the control object from safe region. Turning our attention to Fig. 6(b), it is evident that the traditional ${H}_{\infty}$ ADP scheme still fails to ensure safety under random disturbance. It is, however, worth noting that the proposed scheme requires more than one iteration to establish a safe policy. This is because during the training process, especially for the initial phases of training, even though the iterative control policy is to train against the disturbance policy targeted at maximizing the performance function, the iterative disturbance policy is not the worst one. This means that the given random disturbance could be worse than the disturbance in the current iteration. Consequently, the improved policy may not be able to cover the disturbances that are worse than those encountered within the first iteration. As iterations progress, this situation would be mitigated, thereby forming a gradual strengthening of the proposed scheme’s robustness.

**Fig. 7:**
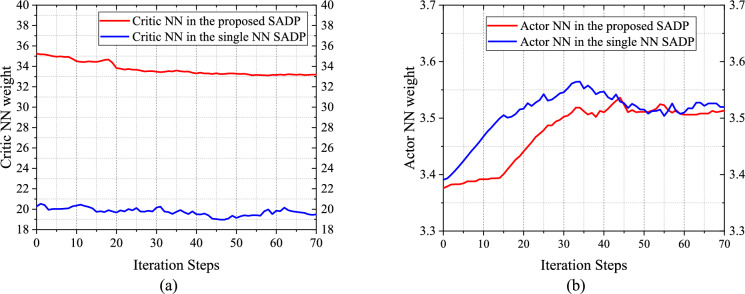
Convergence trajectories of NN weights. **a** Critic NN weights $\Vert {\widehat{W}}^{c}\Vert$. **b** Actor NN weights $\Vert {\widehat{W}}^{a}\Vert$

To validate that alleviating the underestimation of terminal performance function can bring positive effects for training, the dual critic NNs and single critic NN schemes are compared, and the results showing the weight norm convergence process of actor and critic NNs are presented in Fig. 7. The weight convergence behavior of the proposed SADP that possesses dual critic NNs is faster and smoother than that with single critic NN. This is because the evaluation result of the iterative control policy with dual critic NNs can be more accurate, which can provide a better direction for PIM. Therefore, the more optimal result can be achieved even with faster convergence process.

**Fig. 8:**
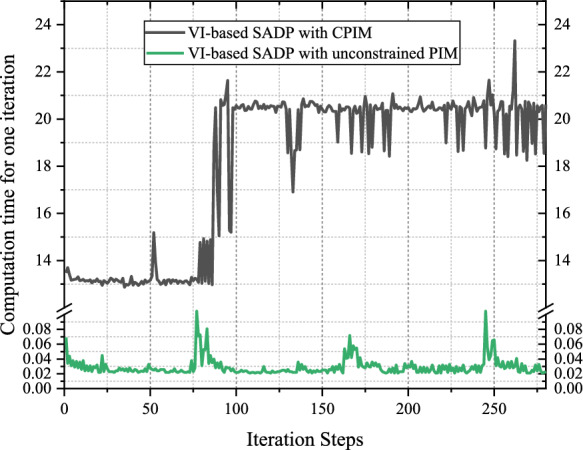
The one iteration computation times of VI-based SADP with constrained PIM and unconstrained PIM

**Fig. 9:**
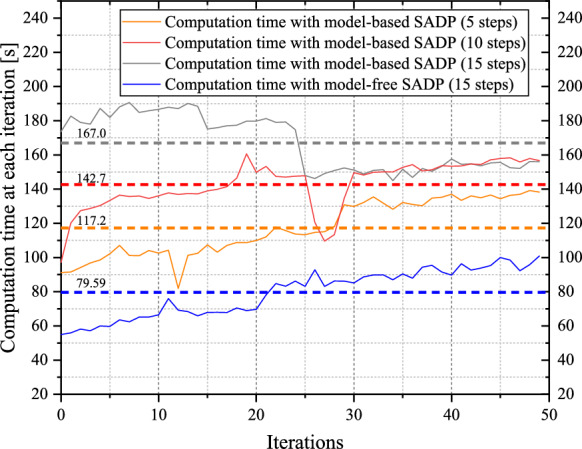
Computation time at each iteration with model-free and model-based schemes

The off-policy multi-step SADP based on VI scheme is compared with the proposed PI SADP scheme. In fact, if the constrained PIM process is considered into the VI-based scheme, the computation time for each iteration is time-consuming. As shown in Fig. 8, the computation time for VI-based SADP with constrained PIM process is significantly larger than that with unconstrained PIM process [43, 44]. In addition, depicted in Fig. 8 and Fig. 9, although the single iteration time consumed in the VI method is superior to that of the PI method, the VI method cannot base its iterations on an admissible strategy, resulting in significantly more iterations compared to PI under constrained PIM. Therefore, the total training consumption of PI is less than that of VI.

For the realization of the multi-step off-policy SADP scheme, there are two ways to be presented in this study, i.e., model-free and model-based schemes. In fact, the essential difference between the two schemes relies on the way of collecting data, in which one is to collect data via interacting with environment, and another one is to utilize dynamics model information. In Fig. 9, the computation time of each PI loop with the two schemes is presented. To compare the computation time quantitatively, the average time of the two schemes is calculated, in which that of the model-free scheme is 79.59 s, yet the model-based scheme should consume 117.2 s (5 steps), 142.7 s (10 steps), and 167.0 s (15 steps) that are all slower than the model-free scheme. Nonetheless, even though the model-based scheme is slower than the model-free scheme obviously in the perspective of the simulation solving speed, whether the solving procedure can be completed within each sample time is an open problem worthy challenge in practical applications, which is the key factor to realize the model-free scheme in real applications. Besides, in the case of difficulty collecting high quality data, the model-based scheme can be particularly significant. Therefore, both model-free and model-based schemes should be developed simultaneously.

## Experiment

### Platform introduction

**Fig. 10:**
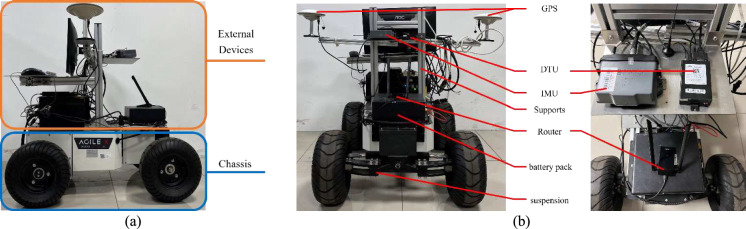
The UGV platform. **a** The side view of UGV platform. **b** The front view of UGV platform.

This section introduces an experiment platform, a programmable HUNTER 1.0 unmanned ground vehicle (UGV), which exhibits driving characteristics similar to real-world autonomous vehicles. This platform possesses flexible functional extensibility, allowing users to install various types of sensors and external devices according to actual task requirements, such as cameras, LiDARs, global positioning systems (GPS), inertial measurement units (IMU), data transmission unit (DTU), and industrial personal computer (IPC). Practical tests can be conducted to validate the effectiveness of proposed algorithms based on the HUNTER 1.0 platform. Subsequently, algorithm performance can be further evaluated and enhanced based on the analysis results of the tests, which provides an important practical foundation for the development and optimization. Different perspectives of the HUNTER 1.0 platform are shown in Fig. 10.

The core function of this chassis is to execute upper-layer control algorithms transmitted via the CAN bus. The construction of the UGV platform and the algorithm design fully consider the parameters of the vehicle chassis, as shown in Table 1.

**Table 1:** The parameters of the UGV platform

Type	Name	Parameters
Mechanical parameter	Length $\times$ Width $\times$ Height(mm)	978 $\times$ 721 $\times$ 330
	Wheelbase (mm)	650
	Driving motor for momentum (W)	DC brushless 2 $\times$ 200
	Driving motor for steering (W)	DC brushless 200
	Maximum steer angle ($^\circ$)	25
	The accuracy of steering ($^\circ$)	0.5
	Drive	Rear wheel drive
	Steer	Front-wheel Ackerman
Performance parameter	Maximum speed without load (m/s)	1.5
	Maximum gradeability ($^\circ$)	20

### Experiment test

In this section, a practical experiment was conducted to validate the proposed SADP method. Specifically, the effectiveness of the proposed approach was verified through offline training and online deployment, where the trained control policy is applied to the UGV platform based on robot operating system (ROS).

For this practical platform, a kinematics model based on the real-axle center is established, expressed as follow

61 $$\left[\begin{array}{c}{X}_{r,t+1}\\ {Y}_{r,t+1}\\ {\varphi}_{veh,t+1}\end{array}\right]={v}_{x,t}\left[\begin{array}{c}0.1\text{cos}{\varphi}_{veh,t}\\ 0.1\text{sin}{\varphi}_{veh,t}\\ \frac{0.1\text{tan}{\delta}_{f,t}}{{{\ell}}_{f}+{{\ell}}_{r}}+{\omega}_{t}\end{array}\right]+\left[\begin{array}{c}{X}_{r,t}\\ {Y}_{r,t}\\ {\varphi}_{veh,t}\end{array}\right]$$

where the state variable is denoted as ${x}_{t}={\left[{X}_{r,t},{Y}_{r,t},{\varphi}_{veh,t}\right]}^{T}$, while the control input can be represented as ${u}_{t}={\left[{v}_{x,t},{\delta}_{f,t}\right]}^{T}$. Specifically, ${X}_{r,t}$ and ${Y}_{r,t}$ represent the position of UGV, and ${\varphi}_{veh}$ is the heading angle. ${v}_{x}$ represents the forward velocity of UGV, and ${\delta}_{f}$ denotes the steer angle of the front wheel.

**Fig. 11:**
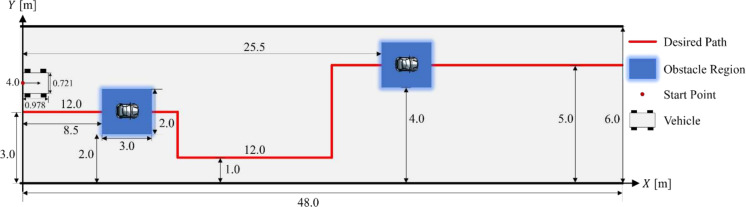
The schematic diagram of the practical experiment based on UGV platform

As depicted in Fig. 11, the specific experimental scenario and control object are defined as follows: The vehicle initializes at a certain point (${x}_{0}={\left[\text{0.0,4.0,0.0}\right]}^{T}$) within the state space. Designing a control law minimizes the given cumulative utility function, while leading the vehicle to safely track the desired trajectory (represented by the red horizontal segments in Fig. 11) within finite time. The feasible state space for this experiment is defined as ${\Omega}_{x}=\left\{{x}_{t}|0\le {X}_{r,t}\le \text{48,0}\le {Y}_{r,t}\le 6,-\pi \le {\varphi}_{veh,t}\le \pi \right\}$, and the action space is defined as ${\Omega}_{u}=\left\{{u}_{t}|-\pi \le {\omega}_{veh,t}\le \pi \right\}$.

For the optimal control problem based on the above experimental scenario, the utility function is defined as $U\left({x}_{t},{u}_{t},{\omega}_{t}\right)={x}_{t}^{T}Q{x}_{t}+{u}_{t}^{T}R{u}_{t}-\varrho {\omega}_{t}^{T}{\omega}_{t}$, where $Q=\text{diag}\left\{0.0,{10}^{-2},{10}^{-2}\right\}$, $R={10}^{-2}$, and $\varrho =0.2$. It is worth noting that the forward velocity of the UGV in this scenario remains fixed at $0.2\text{m}/\text{s}$.

In this case, the unsafe region $\text{Int}\left({\mathcal{C}}_{s}\right)$ is the blue areas shown in Fig. 11. The safe function is composed of the utility function $U\left({e}_{t},{u}_{t},{\omega}_{t}\right)$, the single-step safe function ${J}_{c}\left({x}_{c,t}^{1}\right)$, and the cost function ${J}_{c}^{{\mathbb{D}}_{(\bullet )}}\left({x}_{c,t}^{1}\right)$ for safe region.

Take the first unsafe region as an example, the safe region within the feasible state space ${\Omega}_{x}$ can be presented as

62 $${\mathcal{C}}_{s}={\mathbb{D}}_{1}+{\mathbb{D}}_{2}+{\mathbb{D}}_{3}+{\mathbb{D}}_{4}$$

where

63 $${\mathbb{D}}_{1}=\left\{x\in {\mathbb{R}}^{m}|{x}_{1}\le 8.5\right\}$$

64 $${\mathbb{D}}_{2}=\left\{x\in {\mathbb{R}}^{m}|{x}_{1}\ge 11.5\right\}$$

65 $${\mathbb{D}}_{3}=\left\{x\in {\mathbb{R}}^{m}|8.5\le {x}_{1}\le 11.5,{x}_{2}\le 2.0\right\}$$

66 $${\mathbb{D}}_{4}=\left\{x\in {\mathbb{R}}^{m}|8.5\le {x}_{1}\le 11.5, {x}_{2}\ge 4.0\right\}$$

Based on the safety region ${\mathcal{C}}_{s}$, the safe function is defined, which further incorporates safety-related driving costs on top of the utility function. Specifically, within the non-safety region $\text{Int}\left({\mathcal{C}}_{s}\right)$, a single-step safe function ${J}_{c}\left({x}_{c,t}^{1}\right)$ is set as $1.5$. Additionally, considering the potential discontinuity of the safe function when the vehicle transitions from the safety region to the non-safety region within the state space, a cost function for safety region is further defined as below

67 $${J}_{c}^{{\mathbb{D}}_{(\bullet )}}\left({x}_{c,t}^{1}\right)=\left\{\begin{array}{cc}0& x\in {\mathbb{D}}_{1}\\ 0& x\in {\mathbb{D}}_{2}\\ \text{min}\left(-\text{ln}\left(\frac{-\left({Y}_{r}-0.5\right)\left({Y}_{r}-2.0\right)}{1+\left({Y}_{r}-0.5\right)\left({Y}_{r}-2.0\right)}\right)+0.26,1.5\right)& x\in {\mathbb{D}}_{3}\\ \text{min}\left(-\text{ln}\left(\frac{-\left({Y}_{r}-4.0\right)\left({Y}_{r}-5.5\right)}{1+\left({Y}_{r}-4.0\right)\left({Y}_{r}-5.5\right)}\right)+0.26,1.5\right)& x\in {\mathbb{D}}_{4}\end{array}\right.$$

Thus,

68 $${U}_{c}\left({x}_{c,l}\right)=\left\{\begin{array}{cc}U\left({e}_{t},{u}_{t},{\omega}_{t}\right)+{J}_{c}^{{\mathbb{D}}_{1}}\left({x}_{c,t}^{1}\right)& x\in {\mathbb{D}}_{1}\\ U\left({e}_{t},{u}_{t},{\omega}_{t}\right)+{J}_{c}^{{\mathbb{D}}_{2}}\left({x}_{c,t}^{1}\right)& x\in {\mathbb{D}}_{2}\\ U\left({e}_{t},{u}_{t},{\omega}_{t}\right)+{J}_{c}^{{\mathbb{D}}_{3}}\left({x}_{c,t}^{1}\right)& x\in {\mathbb{D}}_{3}\\ U\left({e}_{t},{u}_{t},{\omega}_{t}\right)+{J}_{c}^{{\mathbb{D}}_{4}}\left({x}_{c,t}^{1}\right)& x\in {\mathbb{D}}_{4}\\ {J}_{c}\left({x}_{c,t}^{1}\right)& x\in \text{Int}\left({\mathcal{C}}_{s}\right)\end{array}\right.$$

Subsequently, to further balance solution efficiency and control performance, a relaxation approach is applied to the safety constraints during a single iteration of training. Specifically, the control policy employed on the UGV platform represents the ultimate training outcome, obviating the necessity to validate each iterative control policy individually. Therefore, validation focuses solely on ensuring that the final result meets the prescribed performance criteria. This relaxation is denoted as follows:

69 $${\left.{J}_{c}\left({x}_{c,t}^{{N}_{c}}\right)\right|}_{\widehat{u}}={\left.\sum_{l=t}^{{t+N}_{c}-1}{\gamma}_{c}^{l-t}{U}_{c}\left({x}_{c,l}\right)\right|}_{\widehat{u}}\le \text{max}\left\{{\left.{J}_{c}\left({x}_{c,t}^{{N}_{c}}\right)\right|}_{\widehat{u}={\widehat{u}}_{i}},{d}_{c}\right\}$$

The implication of the above equation can be indicated as follows: if the control policy fails to drive the system into the safe region, the iteratively improved policy must exhibit superior safety performance compared to the original policy. However, when the control policy satisfies safety requirements, it must consistently maintain the given safety constraints.

**Fig. 12:**
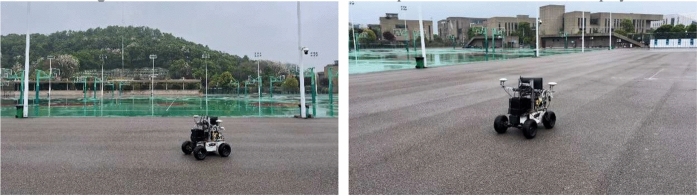
The practical experiment scenario of UGV motion control

Figure 12 represents the real scenario corresponding to Fig. 11. The vehicle shown in Fig. 12 is the control object, which was required to be driven from the starting point, avoid obstacle vehicles, and subsequently converge towards different desired paths. It is worth noting that obstacle vehicles in this case are virtual entities, i.e., the regions of obstacle vehicle are defined on the computer terminal instead of physical vehicles.

**Fig. 13:**
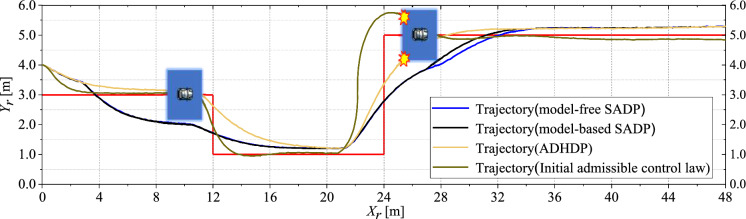
The trajectory of UGV

Before testing in the real-world scenario, the proposed SADP algorithm was trained using PyTorch based on the established simulation scenarios shown in Fig. 11. Subsequently, the trained actor network was deployed on the vehicle’s industrial computer. During the experiment, the control policy was determined by the deployed actor network. The results of the practical experiment are depicted in Fig. 13 They indicate that, compared to the traditional ADP method, the controller obtained through SADP can effectively steer the UGV to avoid hazardous areas and converge towards the desired path. Also, it is evident that the final results obtained by model-based or model-free methods are both feasible in the practical case.

**Fig. 14:**
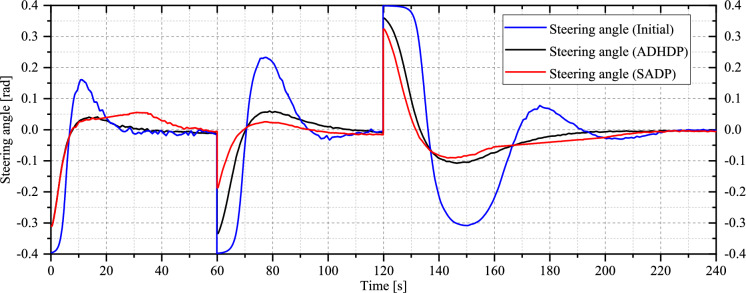
The control behaviors of UGV based on different control law

Corresponding to the result shown in Figs. 13, 14 represents the control behaviors based on different control laws. It is evident that control policies obtained from ADHDP and SADP methods outperform that obtained from the initial admissible control law in terms of magnitude of steering angle.

From the experimental results, it is apparent that there are certain discrepancies between the real-world vehicle test and the desired outcome. Moreover, the control behavior during practical experiments exhibited some fluctuations, likely attributed to positioning errors. Specifically, errors caused by the localization may result in initial calculation errors of the current state of UGV, thereby affecting the inference accuracy of the actor network and consequently hindering precise output of the front-wheel steering angle.

## Conclusion

In this study, a multi-step off-policy SADP was proposed to solve optimal control problems considering safe constraints and disturbances. This approach can employ both model-based and model-free paradigms. To search for the optimal control policy more accurately and feasibly, we introduced an interleaved method and dual critic NNs to alleviate underestimation of the performance function in the model-free approach. For the model-based scheme, the use of prior model information could achieve a similar outcome. After improving the precision of PEV, we also improved the PIM step by transforming the original unconstrained PIM step into a constrained policy optimization problem. Moreover, to manage disturbances when considering safety constraints, we introduced the actor-critic-disturbance architecture and embedded the update of the disturbance policy into the PEV process to guarantee convergence. Finally, simulation results were presented to demonstrate the effectiveness of these methods in terms of safety, robustness, and control performance.

For the model-based multi-step off-policy SADP scheme, the computational burden is non-negligible. It is noteworthy that the implementation of multiple inputs of the critic network is one of the main reasons that lead to the heavy computational burden. Therefore, how to simplify the computation complexity of the model-based scheme is an open issue in our future work.

## Data Availability

No datasets were generated or analysed during the current study.
